# MicroRNA Control of Hepatocyte–Stromal Crosstalk in the Early Premalignant Microenvironment of HBV-Associated HCC

**DOI:** 10.3390/ijms27177581

**Published:** 2026-08-24

**Authors:** Kurt Sartorius, Anna Kramvis, Anil Chuturgoon

**Affiliations:** 1 School of Laboratory Medicine and Molecular Sciences, University of KwaZulu-Natal (UKZN), Durban 4041, South Africa; 2 Africa Hepatobiliary Cancer Consortium (AHPBCC), Mayo Clinic, Jacksonville, MN 55902, USA; 3 Hepatitis Virus Diversity Research Unit, University of the Witwatersrand, Johannesburg 2000, South Africa

**Keywords:** HBV infection, miRNA, hepatocyte, stromal-cell, fibrogenic, angiogenesis, inflammation, CAFs

## Abstract

Chronic hepatitis B virus (CHB) infection remains a major cause of hepatocellular carcinoma (HCC), yet the premalignant microenvironment that links to HBV-associated HCC (HBV-HCC) is still poorly defined. This review synthesizes evidence that HBV-infected hepatocytes function as signaling hubs that, through microRNA (miRNA)-regulated crosstalk with Kupffer cells, liver sinusoidal endothelial cells, hepatic stellate cells and cancer-associated fibroblasts (CAFs), progressively remodel the liver from an antiviral tissue into a premalignant and early tumor microenvironment. Across the HBV-HCC continuum, a core set of dysregulated miRNAs, including miR-21, miR-29a/b, miR-122, miR-146a, miR-155, miR-200a, miR-126, miR-210 and the miR-130/301 family, coordinates transition from innate antiviral responses to HSC activation, extracellular matrix deposition, mechanotransduction, angiogenesis, chronic inflammation and cancer-associated CAF programing. By mapping these stage-specific miRNA networks onto acute infection, CHB, early fibrogenesis, advanced fibrosis and CAF-rich dysplastic states, the review reframes HBV-HCC pathogenesis as a sequence of miRNA-guided hepatocyte–stromal states rather than a purely hepatocyte-intrinsic process. This perspective suggests that composite, cell-type-resolved miRNA signatures in serum or liver tissue could serve as biomarkers for identifying CHB patients who are entering a premalignant microenvironment before conventional surveillance markers become abnormal. It further highlights miRNA hubs that couple antiviral, fibrogenic, angiogenic and CAF-associated signaling as potential therapeutic targets for reprograming the HBV-driven premalignant microenvironment, with the long-term goal of intercepting HBV-HCC development at earlier, microenvironmentally defined stages.

## 1. Introduction

Chronic hepatitis B virus (CHB) infection remains a major global health challenge and a leading cause of advanced fibrosis and HBV-HCC [1]. Despite preventive vaccination and antiviral programs, CHB remains a primary risk factor for HBV-HCC and a global health issue that is associated with liver-related morbidity and mortality [1,2,3,4]. HBV-HCC pathogenesis is commonly attributed to a progressive accumulation of viral and host molecular alterations within infected hepatocytes. However, increasing evidence indicates that HBV-infected hepatocytes act as signaling hubs that actively influence a multicellular community. In CHB-induced pathogenesis these signaling hubs contribute to the dysregulation of a multicellular liver microenvironment consisting of stromal, endothelial, and immune cells that are embedded in the extracellular matrix (ECM) [5,6,7,8]. Unresolved CHB infection, hepatocyte injury, and inflammation, therefore, promote ongoing hepatocyte stromal interaction that initiates progressive stages of the HBV-HCC continuum. These stages include ongoing hepatic stellate cell (HSC) activation, fibrogenesis, ECM remodeling, angiogenesis, and stromal diversification that collectively promote the onset of the premalignant environment (PME) and its progression to HBV-HCC [5,7,8,9,10].

**Figure 1:**
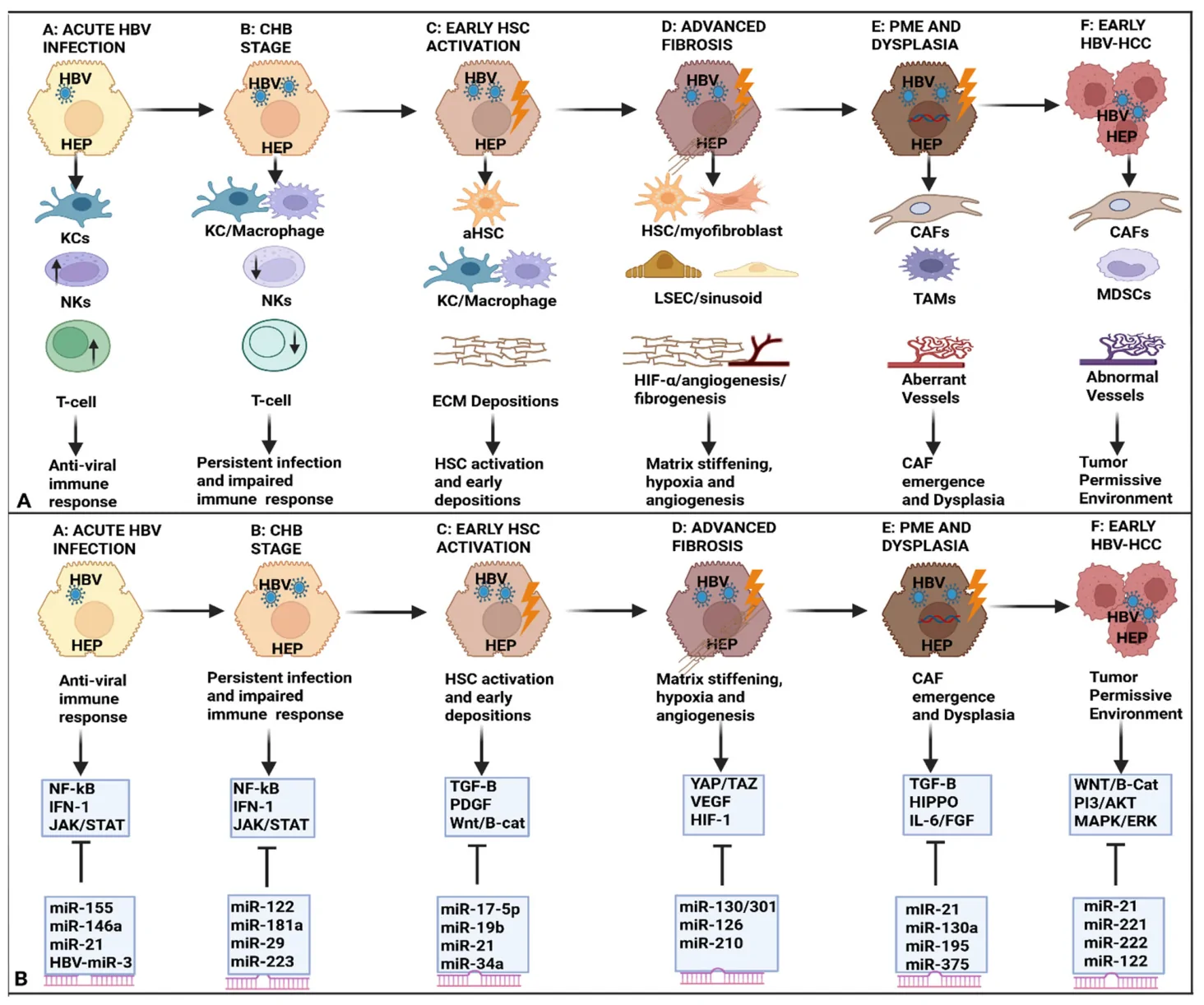
Hepatocyte stromal interaction in HBV=HCC and miRNA regulation of key pathways. (**A**). HBV-infected hepatocytes act as signaling hubs throughout progression from acute HBV infection (A) to chronic hepatitis B (B), early HSC activation and fibrogenesis (C), advanced fibrosis with matrix stiffening and angiogenesis (D), CAF-like stromal-cell emergence and dysplastic nodules within the premalignant microenvironment (E), and early HBV-associated HCC (F). In each stage, the dominant immune, stromal and vascular interactions with hepatocytes are indicated to represent progressive cellular and microenvironmental remodeling rather than specific molecular mechanisms. (**B**). In stages A and B, miR-155, miR-146a, miR-21, HBV-miR-3, miR-122, miR-181a, miR-29 and miR-223 regulate the NF-κB, IFN-I and JAK/STAT signaling during early infection and CHB. In stage C, miR-17-5p, miR-19b, miR-21 and miR-34a regulate TGF-β/SMAD, PDGF and Wnt/β-catenin-dependent to influence HSC activation, proliferation and their fibrogenic responses. In stage D, miR-130/301, miR-126 and miR-210 regulate YAP/TAZ, VEGF and HIF-1α to influence stiffness, angiogenesis and advanced fibrosis. In Stage E, miR-21, miR-130a, miR-195 and miR-375 regulate TGF-β and Hippo signaling to influence the onset of CAFs, fibroblast plasticity and dysplasia. In Stage F, multiple miRNAs like miR-21, miR-221/-222 and miR-122 regulate early HBV-HCC signaling pathways like WNT/B-catenin, PI3K/AKT and MAPK/ERK to influence HBV-HCC pathogenesis. Abbreviations: HEPs (Hepatocytes), KCs (Kupffer Cells), NKs (Natural Killer Cells), aHSCs (Activated Hepatic Stellate Cells), LSECs (Liver Sinusoidal Endothelial Cells, CAFs (Cancer-associated Fibroblasts), TAMs (Tumor-associated Macrophages) and MDSCs (Myeloid Derived Suppressor Cells).

HBV-infected hepatocytes function as dynamic signaling hubs that integrate viral sensing and cellular stress responses by expressing cytokines, growth factors, and extracellular vesicles to communicate with Kupffer cells (KCs), liver sinusoidal endothelial cells (LSECs), hepatic stellate cells (HSCs), fibroblastic stromal cells, lymphocytes, and eventually cancer-associated fibroblasts (CAFs) in the transition to the PME [5,8,10,11,12]. These multicellular interactions progressively alter the liver from an injury repair state to an immune-tolerant, chronic inflammatory fibrotic microenvironment that supports the onset of the PME, dysplasia, and early HBV-HCC [13,14]. Often referred to as an ancillary epigenetic system, microRNAs (miRNAs) regulate hepatocyte–stromal interactions across this transition because they function as post-transcriptional regulators of gene transcription in all cell types and signaling pathways. They, therefore, regulate hepatocyte-intrinsic antiviral programs, stromal and immune cell activation, extracellular matrix (ECM) remodeling, angiogenesis, and fibroblast reprograming [15,16] (Figure 1). For example, in early HBV infection, upregulated miR-146a and miR-155 and downregulated miR-122 regulate innate antiviral and immune responses that activate NF-κB, type I interferon, and JAK/STAT signaling [15,17,18]. By contrast, miR-155 enhances innate antiviral immunity against HBV in hepatoma models, whereas loss of miR-122 promotes HBV replication and weakens hepatocyte innate immune competence [19,20,21]. As HBV infection advances from acute to CHB, HBV proteins like the HBV X protein (HBx) dysregulate these same miRNAs, including miR-146a and miR-122, promoting persistent inflammation, impaired interferon responsiveness, and viral persistence [16,17,21,22]. These changes are not confined to hepatocytes but reshape hepatocyte–immune communication, altering KC, NK, and T-cell function in ways that favor maladaptive repair rather than viral clearance [13,23,24,25,26].

As CHB and hepatocyte damage persist, miRNA-regulated hepatocyte–stromal crosstalk increasingly activates HSCs and promotes fibrogenesis. In activated HSCs, antifibrotic miRNAs such as miR-29a/b are progressively downregulated, removing the repression of collagen depositions and weakening control over TGF/PDGF-associated fibrogenic signaling [27,28,29]. In parallel, upregulated profibrotic miRNAs, including miR-21, miR-17-5p, miR-27a, miR-33a, miR-181b, and miR-221/222, amplify HSC activation, proliferation, and ECM synthesis [30,31,32,33,34,35,36]. Conversely, downregulated anti-fibrotic miR-15a and miR-16, which normally suppress HSC proliferation and fibrogenic depositions, fail to regulate ECM synthesis and the onset of early fibrogenesis [37]. Collectively, these changes promote a shift from transient wound repair toward sustained matrix deposition, tissue stiffness, and the initiation of a fibrotic PME [14,38,39,40].

As liver fibrogenesis advances, specific miRNAs have been mechanistically linked to the interaction between angiogenesis, fibrogenesis, mechanotransduction, and fibroblast reprograming. Upregulated miRNAs, like miR-126 and miR-210, for instance, have been mechanistically validated as being linked to angiogenesis, vasculature modifications, and the influence of hypoxia on LSEC adaptations [41,42,43]. Simultaneously, the upregulated miR-130/310 family can promote YAP/TAZ-dependent profibrotic signaling that interlinks ECM remodeling, mechanotransduction, and persistent stromal activation with changes in the vasculature [44,45,46].

Other upregulated miRNAs, like miR-34a, have also been linked to developmental reprograming and fibrogenesis by TGF-β signaling, while the emergence of CAFs is promoted by upregulated miR-200a targets that enhance Wnt/β-catenin signaling that influences epithelial plasticity, fibrogenesis, and stromal reprograming [47,48,49,50,51]. Once established, CAFs can express miRNA exosomes like downregulated miR-150-3p and upregulated miR-20a-5p that promote hepatocyte migration and the onset of dysplastic hepatocytes [52,53]. In the transition from a PME characterized by advanced fibrosis, dysplastic hepatocytes and CAF-like fibroblasts to the onset of HBV-HCC, an increasing number of dysregulated miRNAs facilitate tumorigenesis. Upregulated oncomirs in dysplastic hepatocytes like miR-21, miR-221, miR-222, and miR-224 repress tumor suppressors and activate PI3K/AKT and mTOR/PAK4 signaling to promote proliferation, survival, and invasive growth [31,54,55,56,57]. Simultaneously, downregulated liver-rich tumor-suppressive miRNAs like miR-122 and miR-199a fail to regulate growth-factor signaling, angiogenesis, and metabolic changes, further promoting the onset of HBV-HCC [20,58,59,60]. Collectively, these examples illustrate how miRNAs participate in the transition from reparative fibrosis into a more angiogenic, mechanically active, and developmentally reprogramed microenvironment that supports CAF emergence and early tumor initiation [8,10,14,45,61,62].

In this review, we illustrate how HBV-infected hepatocytes act as central signaling hubs that communicate with neighboring stromal and immune cells from early HBV infection to the onset of HBV-HCC. We also show how this communication is regulated by miRNAs at each stage of HBV-HCC pathogenesis. In the HBV-HCC continuum, we first show how miRNAs regulate acute-stage antiviral NF-κB, IFN-I, and JAK/STAT signaling in hepatocytes and how they activate neighboring immune cells. We then discuss how miRNAs regulate hepatocyte–stromal interactions in HSC activation, early fibrogenesis, ECM remodeling, vascular reprograming, CAF emergence, and the onset of dysplasia and HBV-HCC. We illustrate that miRNA dysregulation and its regulatory outcomes in HBV-HCC pathogenesis are cell- and stage-specific. A single miRNA can regulate gene expression in multiple targets in hepatocytes, HSCs, macrophages, NKs, LSECs, or CAFs to influence a different biological effect. The direction and degree of miRNA dysregulation are also cell- and stage-specific. We acknowledge a limitation of this paper relates to some of our hypotheses regarding the molecular mechanisms influencing fibrogenesis because these studies used non-HBV models (CCL4). We conclude by proposing that identifying miRNA-regulated hepatocyte–stromal interactions in the HBV-HCC continuum can potentially promote new insights for the development of miRNA therapeutics.

## 2. miRNA Regulation of Hepatocyte–Stromal Interaction in Acute and Chronic HBV Infection

**Figure 2:**
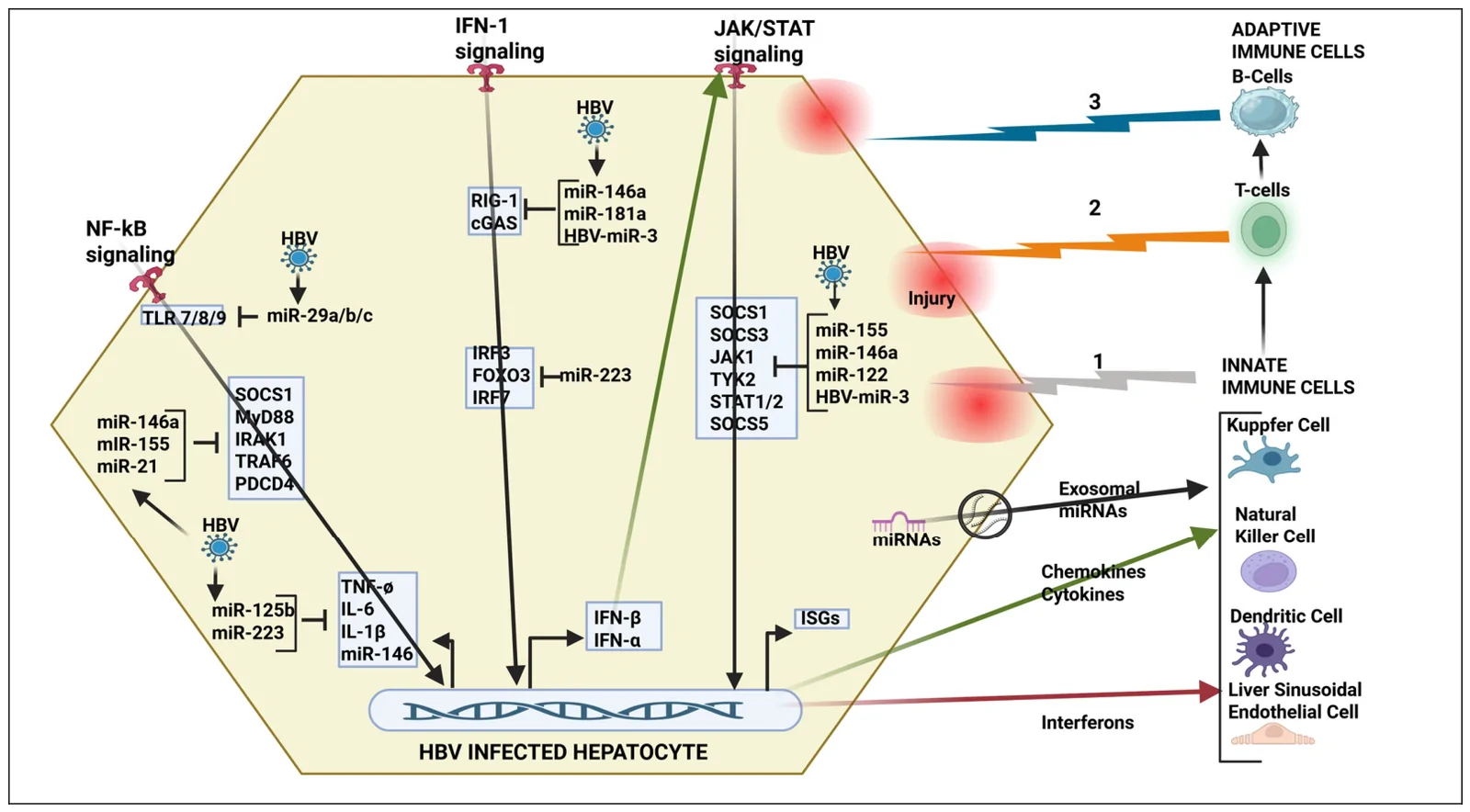
miRNA regulation of antiviral response in HBV-infected hepatocyte signaling hubs. After entry into a hepatocyte, HBV virions are sensed by pattern-recognition receptors that activate NF-κB and IFN-I/JAK-STAT pathways, leading to transcription of cytokines (e.g., TNF-α, IL-6, and IL-1β), type I interferons and interferon-stimulated genes (ISGs). Key miRNAs, including miR-146a, miR-155, miR-21, miR-29a/b, miR-125b, miR-223, miR-181b and miR-122, modulate these cascades by targeting adaptor proteins, negative regulators and STAT-associated checkpoints, thereby tuning intracellular antiviral responses. Dysregulation of these miRNAs by HBV proteins (e.g., HBx/HBsAg) attenuates intracellular anti-viral response and contributes to HBV persistence, innate immune evasion, and low-grade inflammation. Infected hepatocytes secrete cytokines and interferons, as well as exporting miRNA-loaded extracellular vesicles/exosomes that collectively activate innate immune response (KCs, NKs, DCs and LSECs) that re-enforces antiviral response (1) and activates T-cell/B-cell response (2/3) that forms the primary immune response to clear acute stage infection.

Infected hepatocytes in acute-stage HBV infection initially activate an antiviral response in the NF-κB, IFN-I and JAK/STAT signaling pathways that is regulated by a relatively consistent set of miRNAs. These pathways continue to be activated in the transition to CHB. However, ongoing viral replication, injury and inflammation changes the outcome of these same pathways from antiviral defense to immune dysfunction and tissue repair [63,64,65,66]. HBV-infected hepatocytes function as central intracellular and extracellular signaling hubs in acute and CHB stage infection. Initially, infected hepatocytes sense HBV nucleic acids to activate intracellular and paracrine signaling to promote miRNA-regulated inflammatory and interferon responses. These hepatocytes also export cytokines, chemokines and exosomes to activate innate and adaptive immune response from neighboring KCs, NKs, DCs and LSECs that then promote T-cell and B-cell responses [11,12,13]. Figure 2 provides a simplified schematic of this hepatocyte-centric antiviral architecture, highlighting the NF-κB and IFN-I/JAK-STAT pathways and their regulation by key miRNAs that govern both intracellular antiviral responses and paracrine signaling to neighboring immune and stromal cells.

### 2.1. MiRNA-Regulated Hepatocyte Signaling Hubs in HBV Infection

In HBV-infected hepatocytes, viral nucleic acids and proteins are detected by endosomal and cytosolic sensors that converge on the NF-κB and IFN-I/JAK-STAT signaling pathways [65,67,68]. These pathways initiate pro-inflammatory cytokine, chemokine and interferon responses that establish both an intracellular antiviral state and a paracrine signaling network directed toward neighboring immune and stromal cells [63,64,65]. A relatively small group of miRNAs repeatedly regulates these signaling pathways in early and acute-stage CHB including miR-146a, miR-155, miR-21, miR-122, miR-181a, miR-223 and the HBV-encoded miR-3. These miRNAs principally target upstream adaptors, SOCS proteins, STATs or negative regulators to influence the intensity and duration of antiviral signaling [17,19,20,69,70,71,72,73]. Viral proteins, particularly HBx and HBeAg, dysregulate multiple miRNAs like miR-146a, miR-155, miR-21 and miR-122, thereby weakening intracellular and paracrine activation of innate and adaptive immune response and promoting low-grade inflammatory signaling and viral persistence [71,74,75,76,77]. Infected hepatocytes, therefore, act as miRNA-regulated signaling hubs that influence intracellular antiviral response, as well as communicate with neighboring immune cells (Figure 2). Additional important miRNAs regulating acute and CHB stages are included in Table 1.

### 2.2. HBx Modulation of miRNA Expression

Although HBV transcribes a range of proteins that can dysregulate miRNA expression, the HBx protein is the most researched viral transactivator that modulates host miRNA expression to promote HBV replication, persistence, and immune evasion [78,79]. For example, HBx induces miR-146a expression, which attenuates host antiviral response to support viral persistence. It also induces miR-21 to promote IL-6/TGF-β1 signaling by suppressing negative signaling regulators like PDCD4 and PTEN to promote hepatocyte survival, proliferation, and oncogenic responses [74,80]. Conversely, the HBx protein can repress protective miRNAs like miR-122, miR-18a, miR-30e, and miR-132 to promote HBV replication or CTGF/P4HA2-associated fibrogenic and oncogenic signaling [81,82,83]. These examples illustrate how HBx-mediated miRNA dysregulation can regulate the hepatic microenvironment to promote its own replication and survival by modulating host cell machinery to influence antiviral defense, chronic inflammation, HSC activation, and HBV-induced pathogenesis from early infection to the onset of HBV-HCC.

**Table 1:** Key miRNAs in acute and chronic HBV infection.

miRNA	HBV	Target	Effect	Reference
**Early/Acute**				
miR-125b-5p	Up	STAT3/LIN28B	Can both promote/reduce viral clearance	[84,85]
miR-146a/b	Up	STAT1/RIG1/ROG-G/TRAF6	Attenuates pro-inflammatory cytokines	[17,69,74]
miR-155	Up	SOCS1/BCL-6/SHIP-1	Promotes pro-inflammatory cytokines	[19,75,86]
miR-181a	Up	cGAS/E2F5/PTEN/FAS/IL-1α	Retards antigen/innate pro-inflammatory	[73,87,88,89]
miR-199a-5p	Up	DDX3	Promotes cccDNA expression	[84,90]
miR-200b/c	Down	MyD88/TLR4/NFIA	Represses innate response	[91,92]
miR-21	Up	PDCD4	Regulates NF-kB signaling	[71,93]
miR-221-3p	Up	TBK1	Regulates P13K/AKT/NF-kB inflammation	[80,94]
HBV-miR-3	Up	cGAS/SOCS5	Regulates IFN-1 and JAK/STAT signaling	[70,73,84,95,96]
miR-122	Up	STAT1/STAT3	Fails to promote Interferon expression	[21,97]
miR-106a	Down	IL-8	Promotes inflammation	[98,99,100]
miR-548	Down	IFN-λ1	Reduces IFN repression/immune response	[101,102]
**CHB**				
miR-122	Down	CCCDNA/pgRNA	Regulates HBV replication and cell cycle	[20,77]
miR-146a	Up	CFH	Immune response /viral persistence in CHB	[22,23,74]
miR-155	Down	SOCS1	Influences T-cell related response/injury	[86,103]
miR-181a	Up	cGAS/STING/HLA-A	Influences IFN-1 and T-cell response	[24,73,102]
miR-29a/b	Down	SMARCE1/TLR7-8/MyD88	Regulates NF-kB and HBV DNA synthesis	[104,105,106]
miR-223	Down	FOXO3/NLRP3	Influences viral persistence/macrophages	[107,108,109]

### 2.3. Acute HBV Infection: miRNA Control of Hepatocyte-Driven Innate Activation

Two centrally important miRNAs regulating inflammation in HBV infection are miR-146a and miR-155, often described conceptually as a “brake and accelerator” for inflammatory signaling [74,110,111,112]. Typically, the HBV X protein (HBx) can activate NF-κB and upregulate the anti-inflammatory miR-146a, which then dampens Toll-like receptor and NF-κB signaling by targeting the IRAK1 and TRAF6 kinases [74,110]. Conversely, HBx-induced miR-146a can also repress complement factor H (CFH), a negative regulator of complement-driven inflammation, thereby demonstrating that it can exert both an anti-inflammatory and a pro-inflammatory influence in HBV development [74]. Beyond these effects, NF-κB-induced (and HBV-induced) miR-146a can attenuate type I interferon (IFN-I) production in part by targeting the RIG-I sensor and associated adaptor components within the RIG-I pathway [17]. In hepatocytes, miR-146a additionally targets STAT1, thereby blunting IFN-induced activation of the JAK/STAT signaling pathway and impairing the anti-HBV response [113].

In contrast, the pro-inflammatory miR-155 generally promotes NF-κB-driven cytokine production and can enhance antiviral JAK/STAT signaling by repressing SOCS family inhibitors in diverse innate-immune contexts [111,112]. In chronic HBV infection, however, miR-155 is downregulated in NK cells and other compartments, contributing to impaired effector function and facilitating viral persistence [86,111,112]. Experimental overexpression of miR-155 in HBV-infected hepatoma cells enhances antiviral innate immunity, at least in part by boosting IFN-related responses, supporting its role as an “accelerator” of antiviral inflammation in this setting [19]. Other miRNAs modulating multiple innate-immune pathways include HBV-miR-3 and miR-223. The HBV-encoded HBV-miR-3 can attenuate IFN-I signaling and promote immunosuppression by repressing the cGAS cytosolic DNA sensor [70] but can conversely promote antiviral JAK/STAT signaling by repressing SOCS5, which regulates JAK1/2 [96,114]. Finally, downregulated miR-223 in HBV-related liver injury can enhance inflammation by failing to restrain the NLRP3 inflammasome in the NF-κB pathway [108,115], while miR-223 can also promote IFN-I production by targeting the negative regulator FOXO3 [109], collectively positioning miR-223 as an important modulator at the intersection of NF-κB and IFN signaling in HBV disease. In parallel, loss or functional inhibition of liver-enriched miR-122 weakens interferon-associated control of HBV replication and contributes to reduced antiviral efficiency in infected hepatocytes [20,21,97]. This intracellular signaling promotes an antiviral response, as well as the export of cytokines, chemokines, interferons, and miRNA-loaded extracellular vesicles into the hepatic microenvironment [107,116,117]. This paracrine signaling activates neighboring KCs, DCs, NKs, and LSECs, which then amplify antiviral defense through antigen presentation, cytokine secretion, and support of downstream T- and B-cell priming [24,118,119]. Thus, in the acute stage, miRNA regulation of hepatocytes is functionally inseparable from the activation of neighboring innate immune cells, because it determines the intensity and quality of the signals that initiate antiviral immunity across the sinusoidal niche [13,19,21,120].

### 2.4. Transition to Chronic HBV: Persistent Inflammation, Immune Dysfunction and Early Stromal Activation

Many of the same miRNAs that regulate NF-κB and IFN-I–JAK/STAT signaling in innate immune response during the early/acute stage of HBV infection continue to regulate these pathways in the CHB stage. However, in the CHB stage, their regulatory role changes to induce an increasingly proviral, anti-inflammatory pro-survival microenvironment [74,86,121,122]. Dysregulated miR-146a, for example, represses STAT1 to attenuate JAK/STAT antiviral signaling to promote anti-immune type responses [113], as well as repressing STAT1 signaling in T cells and monocytes to blunt their responses [122,123]. In CHB, dysregulated miR-155 and miR-122 also contribute to impaired interferon responsiveness, immune exhaustion, and persistent low-grade inflammatory signaling, thereby linking viral persistence to cumulative hepatocyte injury [20,22,74]. In NK cells, for example, downregulated miR-155 fails to regulate SOCS1, contributing to a decrease in IFN-γ expression in the CHB stage [86]. Various studies indicate that the downregulation of miR-122 in CHB reduces the regulation of targets like cyclin G1, thus failing to provide a brake on persistent viral replication [20,121,124]. HBV-miR-3 is also hypothesized to promote immune evasion because it continues to repress IFN-I/JAK-STAT pathways; however, this needs to be mechanistically verified in a CHB model [96,114]. Similarly, miR-223 also plays an important role in CHB-induced persistent inflammation by targeting the NF-kB pathway and the NLRP3 inflammasome, as well as regulating IFN-1 signaling by targeting FOXO3 [108,109].

Collectively, these examples illustrate that the same miRNAs can promote different outcomes in acute-stage infection versus the CHB stage, which no longer supports efficient viral clearance; instead, it promotes chronic activation or dysfunction of neighboring KCs, macrophages, NKs, and LSECs while also initiating paracrine cues that begin to activate HSCs [13,22,74]. The emerging role of miR-29a/b/c provides an important mechanistic bridge to Section 3 because, although these miRNAs are still linked to inflammatory and antiviral signaling during CHB, their progressive loss linked to the activation of HSCs and the initiation of fibrogenesis [27,105,106].

In this section, miR-155 illustrates stage- and cell-specific effects that not only influence its degree and direction of dysregulation but also its downstream regulatory effects. For example, upregulated miR-155 in acute-stage HBV-infected hepatocytes and macrophages promote NF-κB- and interferon-associated signaling that promotes inflammatory cytokine production and antiviral activity. Conversely, downregulated miR-155 in NK cells during CHB-stage infection allows SOCS1 repression of IFN-γ expression, influencing impaired cytotoxic function and viral persistence [19,75,86].

## 3. The Role of miRNAs in CHB-Induced HSC Activation and Early Fibrogenesis

**Figure 3:**
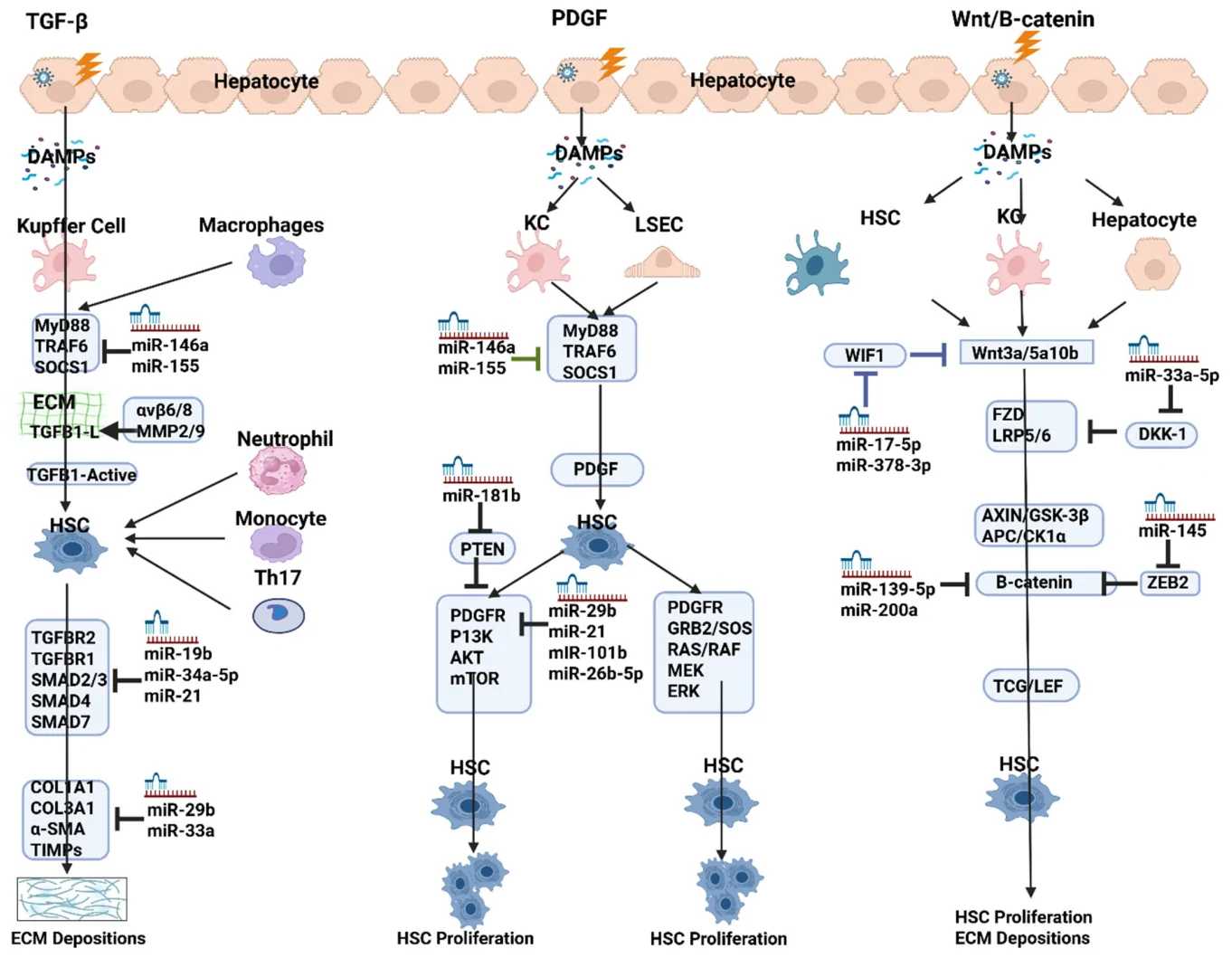
miRNA regulation of hepatocyte–stromal interaction in HSC activation and early fibrosis. This figure highlights three major signaling axes and their regulation by microRNAs (miRNAs). In the TGF-β/SMAD pathway, ligand derived from hepatocytes, KCs and HSCs binds TGF-β receptors on HSCs to activate SMAD2/3–SMAD4 complexes and induce transcription of α-SMA, COL1A1 and other fibrogenic genes. Pro-fibrotic miRNAs such as miR-21 and miR-33a enhance this response by targeting inhibitory regulators (e.g., SMAD7), whereas anti-fibrotic miRNAs such as miR-29 family members suppress collagen gene expression and are progressively down-regulated as CHB-associated fibrogenesis advances. In the PDGF pathway, PDGF-BB released from activated platelets, endothelial cells and macrophages binds PDGFR-β on HSCs to stimulate PI3K/AKT and MAPK signaling that promotes HSC proliferation, migration and survival; miR-21 and miR-181b support this mitogenic signaling by repressing PTEN and cell-cycle checkpoints, while miR-26b, miR-101b and miR-223 act as brakes by targeting PDGFR-β or downstream kinases. In the Wnt/β-catenin pathway, Wnt ligands produced in the chronically inflamed liver activate Frizzled/LRP receptors on HSCs, stabilizing β-catenin and driving transcription of genes that reinforce the activated phenotype; miR-17-5p and miR-27a promote this signaling by inhibiting Wnt antagonists such as WIF1, whereas miRNAs, including miR-29 and other context-dependent regulators, restrain β-catenin activity.

CHB is characterized by repeated cycles of hepatocyte injury, inflammatory signaling, and compensatory tissue repair that progressively reshapes the hepatic microenvironment. In this setting, HSCs become the principal stromal responders to persistent hepatocyte and immune-derived signals, and their activation marks a pivotal transition from antiviral inflammation to fibrogenic remodeling [6,38,39]. In the context of this review, early fibrogenesis is best understood as a stage of miRNA-regulated hepatocyte–stromal crosstalk in which infected hepatocytes, KCs, macrophages, and LSECs collectively drive HSC activation through growth factors, cytokines, extracellular vesicles, and matrix-associated signals [6,26,38]. Figure 3 illustrates how persistent HBV-associated hepatocyte injury stimulates paracrine activation of HSCs through canonical fibrogenic pathways that are regulated by miRNAs to activate HSCs, ECM depositions, and HSC proliferation.

### 3.1. Hepatocyte–Stromal Crosstalk as the Trigger for HSC Activation

Fibrogenesis in ongoing CHB is initiated by injured hepatocytes and their signaling to neighboring stromal and immune cells. Damaged hepatocytes release reactive oxygen species (ROS), mitochondrial DNA, damage-associated molecular patterns (DAMPs), inflammatory cytokines, and growth factors (GFs). Simultaneously, paracrine signaling from injured hepatocytes activates KCs and recruits’ macrophages that express TGF-β, PDGF, and inflammatory mediators to amplify HSC activation [26,38,39,125]. LSECs and platelets further contribute to this signaling environment, illustrating that HSC activation reflects a multicellular hepatocyte/stromal response to unresolved hepatocyte injury [6,38,126]. An important set of miRNAs function as post-transcriptional regulators of HSC activation in the TGF-β, PDGF, and Wnt/β-catenin pathways, including miR-19b, miR-29b, miR-27a, miR-21-5p, and miR-33a (Figure 3). These miRNAs influence the inflammatory and GF expression in the liver microenvironment, as well as directly regulating HSC activation and proliferation, their transcription of ECM materials, and myofibroblast differentiation [31,33,127,128,129].

### 3.2. TGF-Beta/SMAD Signaling and miRNA Control of Fibrogenic Transcription

TGF-β signaling remains the dominant pathway driving the transcription of fibrogenic materials in activated HSCs. Latent TGF-β1 released by macrophages, KCs, HSCs, injured hepatocytes, and endothelial cells (ECs) is activated within the ECM and then binds to TGFBR2/TGFBR1 receptors on nearby HSCs, initiating SMAD2/3/4-induced transcription of COL1A1, COL3A1, and α-SMA [125,130,131,132]. Pro-fibrogenic miRNAs, like miR-21-5p and miR-33a, for instance, promote fibrogenic signaling by repressing SMAD7, which acts as a negative regulator of TGF-β signaling in activated HSCs [27,129,133]. Conversely, the anti-fibrotic miR-29 family can directly repress collagen transcription of COL1A1 and COL3A1 in the TGF-β pathway; however, its expression is downregulated in ongoing CHB-associated fibrogenesis, removing an important restraint on matrix depositions [27,28,29]. Other miRNAs like miR-19b, miR-34a-5p, and miR-122 have also been linked to TGF-β/SMAD-associated regulation of HSC activation or collagen transcription. In HBV-induced fibrosis, for example, downregulated anti-fibrotic miR-34a-5p fails to regulate the TGF-β1/Smad3 pathway; however, in other cases, some of these mechanisms still require stronger validation in HBV-specific models [47,127,134,135]. In CHB, persistent activation of this pathway eventually converts an injury repair response into sustained ECM depositions and early fibrogenesis. In the context of this review, miRNA regulation of hepatocyte–stromal interaction to promote persistent TGF-β signaling can promote the initiation of early fibrogenesis. Figure 3 illustrates miRNA regulation of hepatocyte–stromal crosstalk that activates HSCs and promotes their proliferation and expression in the TGF-β, PDGF, and Wnt/β-catenin signaling pathways.

### 3.3. PDGF Signaling and miRNA Regulation of HSC Proliferation

Contrasting the principal HSC signaling pathways, the PDGF signaling pathway is regarded as the primary driver of HSC proliferation, whereas TGF-β signaling largely promotes the transcription of ECM depositions. In CHB-induced fibrogenesis, ongoing hepatocyte injury and inflammatory activation stimulate KCs, ECs, and platelets to release PDGF ligands, especially PDGF-BB, which activates PDGFR-β on HSCs and triggers signaling in the PI3K/AKT/mTOR and MAPK pathways to promote proliferation, migration, and survival [38,39,126,136]. In this way, ongoing hepatocyte injury promotes both a larger stromal pool and increased transcription of ECM depositions. Several miRNAs influence PDGF signaling in this pathway to influence HSC proliferation. These miRNAs include miR-181b and miR-21 that promote HSC proliferation by repressing PTEN, p27, and related inhibitory checkpoints in PI3K/AKT-associated signaling, thereby attenuating cell division controls to influence the synthesis of activated HSCs [30,35,137,138]. Conversely, anti-fibrotic miRNAs such as miR-26b-5p, miR-101b, and miR-223 restrain PDGF pathway signaling by targeting the PDGFR-β receptor to attenuate downstream HSC proliferation and ECM materials transcription [139,140,141,142]. The increasingly downregulated anti-fibrotic miR-29 family also represses PDGF signaling but progressively influences less control in CHB-induced HSC proliferation and ECM accumulation [28,29,105]. In summary, signaling in the PDGF pathway is increasingly influenced in ongoing CHB by miRNA-regulated hepatocyte and immune cell growth signals that promote stromal proliferation and fibrogenesis.

### 3.4. Wnt/β-Catenin Signaling to Activate HSC Phenotypes

The Wnt/β-catenin pathway amplifies pro-fibrogenic signaling in the TGF-β and PDGF pathways by promoting the persistence of activated HSCs. As fibrogenesis progresses in CHB-induced injury and inflammation, Wnt/β-catenin signaling is induced to “permanently” activate HSC phenotypes, thus amplifying ECM depositions and promoting the emergence of tissue remodeling. CHB-induced apoptosis, ROS and macrophage activation promote Wnt ligand release to engage Frizzled/LRP receptors on HSCs that influence the inhibition of the APC β-catenin destruction complex. This promotes nuclear β-catenin/TCF-LEF transcription that amplifies HSC proliferation and fibrogenic programing [126,143,144,145]. Key miRNAs, such as miR-17-5p and miR-27a, can promote fibrogenesis in Wnt/B-catenin pathway by repressing WIF1 or APC-related control points to enhance β-catenin-dependent HSC activation and expression of α-SMA and collagen genes [32,33]. Conversely, miR-378a-3p, miR-33a-5p, miR-139-5p, miR-200a, and miR-34a have been reported to suppress Wnt ligands and other β-catenin pathway modulators [49,146,147,148,149]. The anti-fibrotic miR-34a, for example, attenuates WNT1 expression; however, its influence is reduced because this miRNA has been reported as downregulated in HBV-induced liver fibrosis [47]. It also should be noted that many of these hypotheses were tested in non-HBV fibrosis models; however, these studies support the broader point that miRNA networks shape how activated HSCs interpret regenerative and developmental signals within the chronically injured liver.

### 3.5. Recurring miRNA Patterns in Early Fibrogenesis

Several recurring miRNA patterns occur across the pro-fibrogenic TGF-β, PDGF, and β-catenin signaling pathways. Upregulated pro-fibrogenic miRNAs, particularly miR-21, miR-17-5p, miR-27a, miR-33a, and miR-181b, typically promote HSC activation by repressing inhibitory regulators such as SMAD7, PTEN, APC, PPAR-γ, or cell-cycle checkpoints. Collectively, the repression of these negative regulators and controls amplifies TGF-β, PDGF, and Wnt-associated fibrogenic signaling [31,33,35,129], whereas downregulated anti-fibrogenic miRNAs, like the miR-29 family, miR-19b, miR-34a-5p, and miR-101b, fail to restrain collagen synthesis, HSC proliferation, or pro-fibrotic transcription [28,29,47,127]. Interestingly, these miRNAs all regulate the interface between hepatocyte damage, inflammation, and stromal/immune responses to emphasize the central focus of this review. The onset of fibrogenesis, therefore, is not simply the consequence of chronic inflammation; it is the result of a miRNA-shaped communication network that progressively converts finite stromal injury repair programs into the permanent activation of the fibrogenic microenvironment. Most miRNA hypotheses in HBV-induced fibrogenesis studies to date, however, were developed by adopting a two-step process. First, the direction of miRNA dysregulation was established in CHB serum or tissue and, second, the expression and outcome of the same miRNA were determined in a non-HBV (CCL4) model, thus assuming the same molecular mechanism would occur in HBV-induced fibrogenesis. It should be clarified, therefore, that experimental evidence of the role of HBV-induced fibrosis is required for multiple miRNA models (Supplementary Table S1). In summary, Section 3 illustrates a conceptual continuum in HBV-induced pathogenesis in which ongoing HSC activation and ECM depositions begin to promote liver stiffness.

## 4. miRNA-Regulated Hepatocyte–Stromal Signaling in ECM Remodeling and Advanced Fibrosis

This stage of the HBV-HCC continuum is characterized by a continuous cycle of ECM accumulation, matrix reorganization, and progressive tissue stiffening that changes the physical and signaling properties of the stromal microenvironment. Canonical pathways that were activated during early fibrogenesis by TGF-β, PDGF, and Wnt/β-catenin signaling continue to promote collagen synthesis, HSC proliferation, and myofibroblast expansion [5,150]. As fibrosis advances, this ongoing signaling is amplified by mechanotransduction and hypoxia-associated signaling, as well as aberrant repair programs that remodel the PME [14,39]. As this transition progresses, miRNAs continue to function as important regulators of all hepatocyte–stromal communication, including the activation and transcription of HSCs and fibroblastic cells. They also regulate signaling responses by infected hepatocytes, immune cells, and stromal populations, as well as chronic inflammation, matrix-derived signaling, and cellular reprograming. Table 2 summarizes the important miRNAs that have been associated with ECM remodeling, mechanotransduction changes, developmental reactivation, and advanced fibrosis.

### 4.1. Stromal–Hepatocyte Interaction in ECM Accumulation and Remodeling

As CHB progresses toward advanced fibrosis, persistent activation of HSCs and myofibroblasts promotes ongoing ECM accumulation in response to progressive tissue damage. Unlike early fibrogenesis, where wound-healing responses are still reversible, advanced fibrosis is characterized by sustained activation of TGF-β/SMAD, PDGF and Wnt/β-catenin pathways that promote ongoing collagen synthesis, myofibroblast expansion and ECM depositions [27,28,105,177]. These ongoing processes are maintained because chronically injured hepatocytes continue to supply inflammatory and profibrogenic signals that reinforce hepatocyte–stromal communication.

Several important miRNAs are dysregulated to promote CHB-induced fibrogenesis. For example, the antifibrotic miR-29 family becomes progressively downregulated in advanced fibrosis, thus contributing to the removal of collagen gene controls and TGF-β/PDGF fibrogenic signaling [27,28,29,105]. Similarly, the reduced expression of antifibrotic regulators such as miR-19b weakens the suppression of collagen synthesis and TGF-β signaling to promote ongoing ECM accumulation [47,127]. Upregulated profibrogenic miRNAs like miR-21, miR-17-5p, miR-27a, miR-33a and miR-221/222 promote ongoing HSC proliferation, survival and ECM production by repressing controls like SMAD7/PTEN or activating pro-fibrotic signaling in the Wnt/β-catenin pathway [32,33,34,93,129,133,178,179,180]. Collectively, these changes promote a transition from regulated tissue repair to persistent ECM depositions and remodeling. The effect of other GFs, like connective tissue growth factor (CTGF), that amplify TGF-β, PDGF, and Wnt/β-catenin-induced ECM depositions are also regulated by miRNAs. Anti-fibrotic miRNAs, like miR-132 and miR-214-5p, can attenuate CTGF promotion of fibrogenesis; however, their effect on persistent CHB-induced fibrogenesis is counterbalanced by a set of profibrotic miRNAs like miR-21-5p, miR-223, and miR-27a that promote CTGF expression [31,33,157,160,161,181,182]. Collectively, these factors promote an imbalance between the production and degradation of ECM depositions that promote physical changes in tissue structure and stromal cell communication.

### 4.2. Mechanotransduction and Reinforcement of the Fibrotic Niche

As CHB-induced fibrosis advances, continued ECM deposition is accompanied by matrix cross-linking, septal formation, and progressive liver stiffening. These physical changes alter integrin-mediated signaling and cytoskeletal tension in hepatocytes, HSCs, and other stromal cells, thus transforming the ECM from a passive support structure into an active signaling platform [6,150]. Simultaneously, the suppression of upstream MST1/2-LATS1/2 kinases activate Hippo YAP/TAZ signaling to promote a mechanically altered environment that supports fibroblast survival, proliferation, and ECM production using mediators like CTGF and CYR61 [61,62,183,184]. These changes create a feed-forward circuit in which HBV-driven inflammation initiates fibrosis, fibrosis increases matrix stiffness, and stiffness further amplifies YAP/TAZ-dependent stromal activation.

Several miRNAs participate in this mechanosensitive phase. The upregulated miR-130/301 family, for example, acts as an amplifier of stiffness-responsive signaling by repressing VGLL4 to enhance YAP/TAZ-dependent profibrotic transcription. These mechanisms, however, have largely been defined in non-HBV systems; nevertheless, they provide a useful framework for understanding how miRNAs could sustain matrix-dependent stromal activation in CHB [44,45,46]. In parallel, upregulated miR-21 indirectly reinforces mechanotransduction by sustaining TGF-β/SMAD-associated fibrogenic signaling, and downregulated miR-29a/b removes regulation of collagens and other ECM materials, collectively contributing to increasing rigidity and mechanotransduction changes in the microenvironment [27,29,31,93,180,185]. In summary, mechanotransduction is, therefore, not a separate process from fibrogenesis but a later stage that is promoted by miRNA dysregulation that promotes persistent biochemical injury signals, thereby ensuring an irreversible advanced fibrotic state.

### 4.3. Developmental Reactivation and Stromal Plasticity

As CHB-induced fibrosis becomes more advanced, chronically injured liver tissue begins to reactivate embryonic-like developmental signaling programs to initiate tissue remodeling and repair. Signaling pathways like Wnt and Hedgehog can be activated to promote plasticity in hepatocytes, HSCs, and fibroblast-like stromal cells. These developmental features favor progenitor-like features, altered differentiation states, and epithelial–mesenchymal transition (EMT) phenotypes [50,186,187]. As a prelude to the onset of malignancy, these changes establish plasticity in the PME in a loss of differentiation in stromal and epithelial cells. This transition is regulated by several important miRNAs. The miR-200 family can attenuate developmental reactivation by repressing ZEB1/2 and CTNNB1, thereby attenuating Wnt/β-catenin-induced plasticity and EMT-related features in hepatocytes and stromal cells [50,186,187]. Similarly, miR-145 targets ZEB2 to illustrate how miRNA networks can attenuate developmental reactivation and mesenchymal transitions in HSCs and related stromal populations [188]. Conversely, pro-fibrogenic miR-34a promotes developmental reprograming and fibrogenesis by repressing SIRT1/p53 controls [51]. In this section, miR-34a and miR-200a illustrate stage- and cell-specific effects that not only influence its degree and direction of dysregulation but also its downstream regulatory effects. In some HSC and liver-fibrosis models, miR-34a-5p acts as an anti-fibrotic agent by repressing TGF-β1/SMAD3 or WNT1-associated signaling. However, viral proteins (HBx) can downregulate this miRNA to attenuate this anti-fibrotic effect that restrains HSC activation and ECM production [47,149]. Conversely, miR-34a can act as a pro-fibrotic agent in HSCs by attenuating SIRT1/p53- or TGF-β-associated pathways [48,51], indicating that these studies represent different stages of disease that influence the expression of HSCs and hepatocytes. A further factor influencing the context dependence of this miRNA could also have been influenced by using differential non-HBV-fibrosis models [47,48,51]. Similarly, the regulatory effect of miR-200a displays cell- and stage-specific context dependence on different epithelial models. This miRNA can repress B-catenin expression to attenuate epithelial cell plasticity by repressing ZEB1/ZEB2. However, this miRNA is also associated with HSC activation in TGF-β-stimulated HSCs and, during later stages, influences fibroblast diversity and the onset of CAFs [49,50]. This evidence reflects that the regulatory effect of miR-200a in hepatocytes, HSCs, or CAFs can influence HBV-HCC pathogenesis in different ways, including influencing epithelial plasticity, fibrogenesis or CAF programing [49,50].

In advanced HBV-induced fibrosis, ECM remodeling and stromal plasticity accompany the progressive miRNA-regulated reorganization of the hepatic immune machinery. Ongoing antigen exposure, inflammatory cytokine production, and mechanotransduction changes promote increasingly dysfunctional HBV-specific CD8+ T-cell and NK-cell responses. Simultaneously, this attenuation in immune response is further diluted by an increase in Tregs, MDSCs, and TAMs [13,189,190]. Upregulated PD-1/PD-L1 signaling within a TGF-β-rich fibrotic matrix further attenuates antiviral T-cell activity [13,24]. KCs and recruited macrophages increasingly adopt repair mechanisms like IL-10, TGF-β, VEGF, and MMP mediators that link immune tolerance to ongoing HSC activation, angiogenesis, and ECM depositions [6,8,13,190]. Important miRNAs like miR-146a continue to regulate NF-κB and interferon-associated inflammatory responses by targeting IRAK1/TRAF6 and STAT1 to contribute to immune tolerance in KCs and diminished antiviral immune responses [17,22,23], while miR-155 regulates macrophage inflammatory output and NK-cell activity to promote impaired antiviral response [19,75]. Other miRNAs, like miR-223 that target NLRP3/FOXO3, influence myeloid-cell inflammatory regulation, as well as influence HSC activation by targeting PDGF and TAZ/IHH/GLI2-associated pathways [108,109]. In parallel, miR-21 and miR-221/222 couple inflammatory persistence to HSC survival, ECM depositions, and fibrogenic signaling [31,36]. Advanced fibrosis, therefore, should be viewed as an immune-fibrotic state in which miRNA interaction with hepatocytes, macrophages, lymphocytes, and activated HSCs promotes a shift from antiviral surveillance to an immune tolerogenic microenvironment [8,13,40].

## 5. Hepatocyte–Stromal Interaction and the Onset of CAFs: The Role of miRNAs

**Figure 4:**
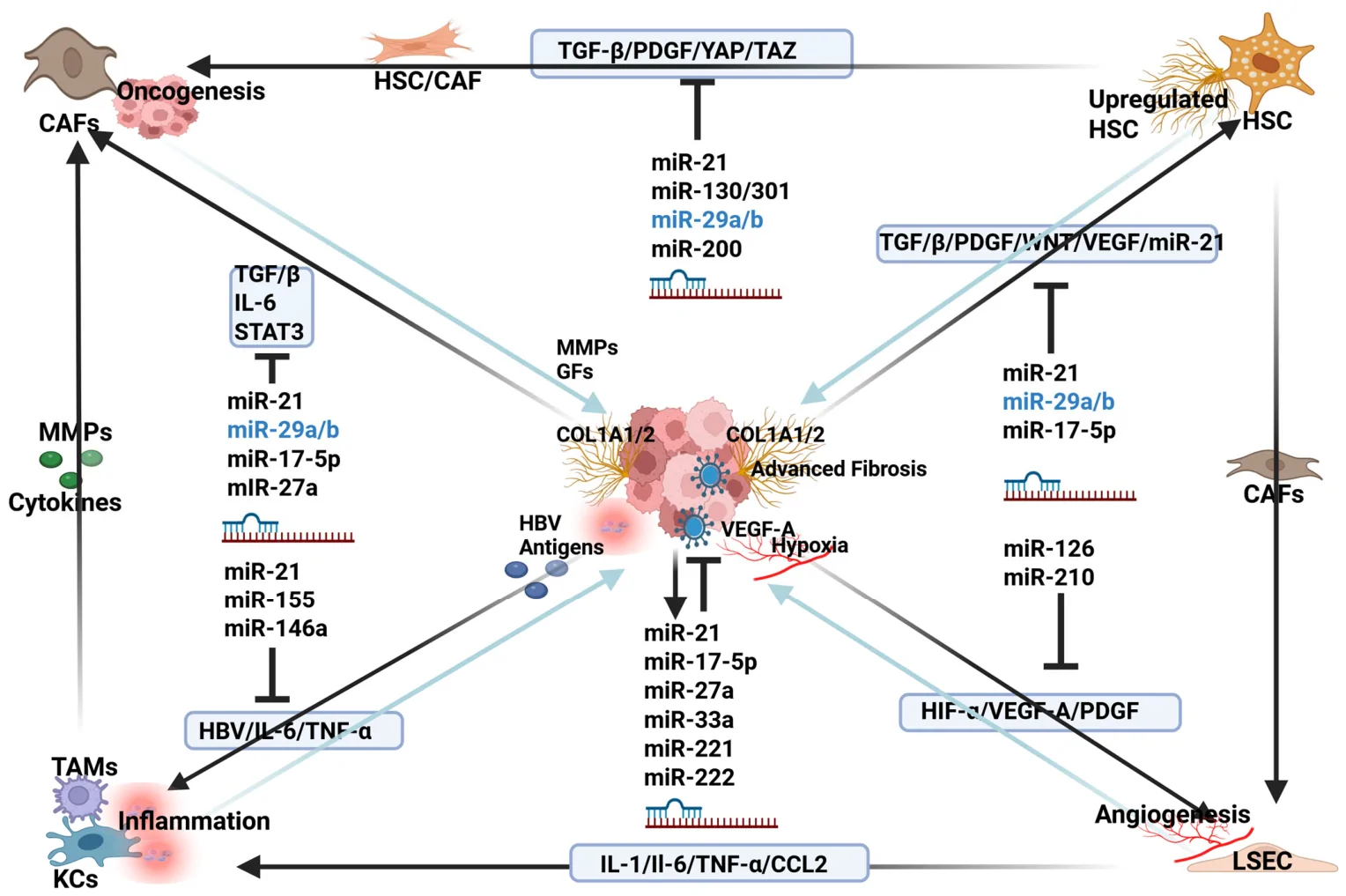
miRNA regulation and the onset of CAFS in HBV-HCC. **Centre:** HBV-infected hepatocytes in an advanced fibrotic liver act as a signaling hub, producing profibrogenic and pro-angiogenic mediators (TGF-β, PDGF, Wnt ligands, VEGF-A, IL-6, and TNF-α) and exporting dysregulated miRNAs (↑ miR-21, miR-17-5p, miR-27a, miR-33a, miR-221/222, and miR-130/301; ↓ miR-29, let-7) in exosomes to surrounding stromal and immune cells. **The top-right** “hepatocyte–HSC axis” shows chronic TGF-β/SMAD, PDGF and Wnt/β-catenin signaling, amplified by these miRNAs and loss of miR-29, driving HSC/portal fibroblast activation, proliferation and ECM accumulation. **The top-left** “HSC–CAF axis” depicts how matrix stiffening, Hippo/YAP–TAZ activation and developmental reprograming, modulated by miR-21, miR-130/301, miR-29 and the miR-200 family, promote HSC transition into CAF-like cells. **The bottom-right** “hepatocyte–LSEC axis” illustrates hypoxia- and HIF-1α-driven VEGF-A/PDGF production by hepatocytes and miR-126/miR-210-regulated VEGF signaling in LSECs, resulting in pathological angiogenesis that supports stromal expansion and dysplastic hepatocyte clusters. **The bottom-left** “hepatocyte–inflammation axis” shows HBV-infected hepatocytes activating Kupffer cells and infiltrating macrophages via antigens and cytokines (IL-6, TNF-α, and TGF-β), whose production and responses are tuned by miR-21, miR-155 and miR-146a, leading to chronic inflammatory–fibrogenic signaling. Stromal–stromal interactions between corners are indicated by arrows: pro-angiogenic, capillarized LSECs promote TAM emergence (LSEC–inflammation axis), whereas KCs/TAMs secrete VEGF, TGF-β, IL-10 and MMPs that reinforce HSC/CAF activation (inflammation–CAF axis). Collectively, the figure summarizes how miRNA-controlled communication along hepatocyte–HSC, HSC–CAF, angiogenic and inflammatory axes drives progressive HSC-to-CAF conversion and formation of a CAF-rich, angiogenic, immunosuppressive premalignant.

The next stage of the HBV-HCC continuum is increasingly characterized by stromal diversification, progressive vascular remodeling, and the emergence of dysplastic hepatocytes and CAF-like stromal cells (Figure 4). At this stage, the PME is transformed from a (hypothetical) reparative state, where the reversal of injury and fibrosis is still possible, to an irreversible, persistent, angiogenic, and immune-tolerant microenvironment that supports the onset of tumorigenesis [8,9,11,12,13,14,61]. This section illustrates how these changes are regulated by miRNA–stromal interaction to emphasize that the emergence of a solid tumor is the result of a community of disorganized cell types rather than aberrant changes in epithelial cells alone. In this microenvironment, persistent signaling from HBV-infected hepatocytes, including cytokines, growth factors, and extracellular vesicles, continues to activate responses in neighboring HSCs, fibroblasts, LSECs, macrophages, and emerging dysplastic hepatocyte clones. Simultaneously, reciprocal signaling from surrounding stromal cells reinforces epithelial plasticity, immune dysfunction, angiogenesis, and fibroblast specialization. Figure 4 should therefore be read as a miRNA-regulated transition from advanced fibrosis to a CAF-rich, dysplasia-supporting premalignant microenvironment.

### 5.1. Advanced Fibrosis and CAF Emergence

The emergence of CAF-like stromal populations is orchestrated in a miRNA-regulated PME that is characterized by ongoing CHB infection and advanced fibrosis. In this transition, profibrogenic miRNAs like miR-21, miR-17-5p, miR-27a, miR-33a, and miR-221/222 are persistently upregulated, and anti-fibrogenic miRNAs like the miR-29 family members and miR-19b remain downregulated. Collectively, these dysregulated miRNAs ensure the ongoing activation of TGF-β/SMAD, PDGF, Wnt/β-catenin, Hedgehog, and Hippo/YAP-associated signaling pathways to promote chronic inflammation, increasing stiffness, and developmentally reprogramed stromal cell types [5,8,14,27,28,30,32,33,34,36,127]. In this setting, permanently activated HSCs and portal fibroblasts acquire increasing signaling features that begin to resemble those of CAFs. The emergence of CAFs, however, should be integrated with the previous section and seen as the next stage of fibrogenic stromal specialization in a microenvironment characterized by persistent matrix stiffening, developmental reactivation, and miRNA dysregulation. Collectively, these conditions shift fibroblastic cells from wound-healing effectors toward immortalized stromal populations that are capable of supporting premalignant hepatocytes [8,14,39,40,61,62].

### 5.2. Angiogenesis and Stromal Reprograming

As CHB-induced fibrosis progresses toward cirrhosis, vascular remodeling becomes increasingly important in shaping the emerging PME. Progressive ECM accumulation, sinusoidal capillarization, and vascular distortion increasingly impair blood flow oxygen supply, creating localized hypoxic regions in the liver. These conditions promote the expression of HIF-1α, VEGF-A, PDGF, and other pro-angiogenic mediators in neighboring LSECs and myeloid cells to promote endothelial proliferation, capillarization, and pathological vascular remodeling that further supports stromal expansion and dysplastic hepatocyte survival [10,11,12,13,15,191]. Several miRNAs regulate angiogenesis and stromal reprograming in this transition. In response to localized hypoxia, miR-210 promotes endothelial adaptation to reduced oxygen supply and endothelial cells express miR-126 to regulate VEGF signaling and vascular integrity by SPRED1 and PIK3R2 [41,42,43]. HBV-induced expression of miR-21 further amplifies TGF-β/SMAD, PI3K/AKT, and inflammatory signaling pathways that promote VEGF production. Simultaneously, downregulated miR-29a/b and miR-19b continue to promote ECM accumulation and vasculature adaptations by failing to regulate collagen depositions and ECM remodeling [27,28,76,127]. Collectively, these changes promote a pro-angiogenic and capillarized microenvironment that supports stromal specialization and the earliest stages of dysplastic progression.

### 5.3. Chronic Inflammation and Reciprocal Stromal Signaling

At this stage of HBV-induced pathogenesis, chronic inflammation is no longer a response to viral presence but a permanently entrenched feature of a fibrotic PME. Reciprocal crosstalk between HBV-infected hepatocytes, KCs, infiltrating macrophages, and HSCs sustain NF-kB/JAK/STAT-dependent production of TNF-α, IL-6, and TGF-β that reinforces stromal activation, angiogenesis, and epithelial stress adaptations [10,13,14,189,190]. In this continuum, permanent inflammatory signaling, ECM remodeling, and fibroblast adaptations are interlocked rather than the original immune-related inflammatory response to the presence of a pathogen.

Chronic inflammation and reciprocal stromal signaling are regulated by several miRNAs expressed by hepatocytes and myeloid cells, like miR-155, miR-146a, and the miR-221/-222 family. Chronic inflammation is enhanced by balancing pro- and anti-inflammatory miRNAs in the NF-kB signaling pathway. The pro-inflammatory miR-155 enhances inflammation and macrophage activation, whereas miR-146a attenuates excessive cytokine production by targeting IRAK1 and TRAF6 [19,69,75,110]. The HBx-upregulated miR-221/222 cluster, on the other hand, contributes to stromal persistence by promoting HSC proliferation, scarring, and chronic inflammation, thereby enhancing growth-factor signaling within the fibrotic microenvironment. This miRNA cluster also influences immune-cell composition by favoring regulatory T-cells (Tregs), myeloid-derived suppressor cells (MDSCs), and tumor-associated macrophages (TAMs) that promote a more tolerogenic and pro-survival microenvironment [36,81,178,192]. Collectively, these miRNAs illustrate how they assist the conversion of chronic inflammatory injury into a stable inflammatory–fibrogenic feedback loop that promotes the emergence of CAFs and the onset of tumorigenesis.

### 5.4. The Onset of CAFs and the Premalignant Microenvironment (PME)

A central hepatocyte–HSC/fibroblast axis persists throughout the transition of HSCs to CAF-like stromal cells. HBV-infected hepatocytes and inflammatory cells continue to release TGF-β, PDGF, Wnt ligands, and other profibrogenic mediators, while dysregulated miRNAs regulate the degree and persistence of stromal responses to this signaling [11,13,14,15]. Upregulated miR-21 amplifies TGF-β/SMAD and PI3K/AKT signaling through repression of SMAD7 and PTEN, while miR-17-5p, miR-27a, miR-33a, and miR-221/222 enhance Wnt/β-catenin signaling, HSC proliferation, and fibroblast survival by targeting regulators such as WIF1, APC, PPAR-α, and CDKN1B/p27 [5,10,30,31,32,33,34,36]. Conversely, the downregulation of miR-122 in the PME, together with other antifibrotic miRNAs such as miR-29 family members, let-7, and miR-19b, removes inhibitory control over collagen synthesis, CTGF-associated signaling, and matrix organization, thereby allowing activated HSCs and fibroblastic cells to remain locked in a chronically activated state [5,8,26,27,28,127].

Persistent exposure of HSCs and portal fibroblasts to TGF-β, PDGF, VEGF, inflammatory cytokines, and matrix stiffness promotes their transition to CAF-like phenotypes. This process is reinforced by mechanotransductive Hippo/YAP-TAZ signaling, in which YAP/TAZ cooperate with SMAD-dependent programs to induce CTGF, ECM proteins, and fibroblast survival pathways, while the miR-130/301 family further amplifies stromal activation through repression of VGLL4 and enhancement of YAP/TAZ transcriptional output [7,24,38,40,44,45,46]. Dysregulation of the miR-200 family also influences epithelial plasticity and stromal communication through ZEB1/ZEB2- and Wnt/β-catenin-associated programs, thereby facilitating fibroblast diversification and early CAF formation [40,49,50].

Within this stiff, angiogenic, and chronically inflamed microenvironment, activated HSCs and portal fibroblasts progressively acquire CAF-like properties, particularly at the edges of dysplastic nodules. This transition is reinforced by persistent hepatocyte-derived cytokines, matrix signals, and extracellular vesicles that reprogram stromal cells away from a wound-healing phenotype toward one that actively supports premalignant hepatocyte clones and the emergence of CAFs [11,14,40]. In the premalignant microenvironment, for example, HBx-induced miR-21 can transform non-tumor hepatocytes by activating IL-6 and TGF-β1 signaling, as well as being exported in exosomes into the neighboring microenvironment to activate HSCs; in turn, ongoing HSC activation promotes their trans differentiation into CAFs [31,76,193]. HSC activation in the HBV-HCC PME is collectively influenced by HBV-infected hepatocytes and neighboring stromal cells that deliver exosomal miRNAs and growth factors such as PDGF, HGF, VEGF, and CTGF [194,195].

The mechanism of HSC upregulation and trans differentiation into CAFs has been described as dysplastic or malignant hepatocytes secreting exosomal miR-21 that represses SMAD7 and PTEN in neighboring HSCs, which also lose vitamin A storage. Together, these changes activate PDK1/AKT signaling and promote HSC survival, thereby driving their differentiation into CAFs [196,197]. It should be noted that trans-differentiated myofibroblasts (CAFs) arising in the HCC TME are generally regarded as permanently activated and “immortalized,” whereas in the PME they are theoretically reversible and may return to a quiescent state or undergo apoptosis if pathogenic signaling is removed [198,199]. In this context, CAFs can be described as trans-differentiated resident liver cells (HSCs/portal fibroblasts) with a persistent myofibroblastic phenotype [200].

At the PME/TME interface, CAFs secrete VEGF, MMP-2, MMP-9, basic FGF, HGF, and TGF-β into surrounding non-tumor tissue to promote growth, angiogenesis, and invasion, illustrating how crosstalk between damaged or malignant hepatocytes and HSC-derived CAFs promotes both CAF emergence and oncogenesis [194,197,200,201,202,203,204,205]. CAFs can also secrete exosomal miR-20a-5p that targets LIMA1 to activate Wnt/β-catenin signaling and initiate tumor cell proliferation, invasion, migration, and EMT [52], and downregulated CAF-derived miR-150-3p can relieve repression of GAB1 to promote RAS/MAPK signaling, invasion, migration, and EMT [53]. These hypotheses still require experimental validation in HBV-HCC models; however, elevated exosomal miR-21 and related dysregulated miRNAs in HBV-associated liver disease, including miR-146a, miR-126, miR-210, and miR-130/301, support the idea that this stromal reprograming begins before overt tumor formation [23,41,42,44].

Collectively, these examples illustrate how CAFs progressively promote oncogenesis in the TME by amplifying hepatocyte transformation and by secreting TGF-β, VEGF, chemokines, and matrix-remodeling enzymes that promote hepatocyte proliferation, epithelial–mesenchymal plasticity, invasive behavior, and immune evasion, while CAF-derived extracellular vesicles and miRNA cargo reinforce pro-tumorigenic signaling in epithelial and immune compartments [1,7,13,40]. Although not all of these mechanisms have been demonstrated specifically in premalignant HBV tissue, they support a model in which miRNA-regulated hepatocyte–stromal communication evolves from driving fibrosis and angiogenesis to establishing a bidirectional signaling circuit between dysplastic hepatocytes and CAF-rich stroma that stabilizes the early HBV-HCC microenvironment.

Taken together, the onset of CAFs can be viewed as the next stage of the HBV-HCC continuum in which advanced fibrosis, angiogenesis, inflammatory persistence, and developmental reprograming converge. Persistent hepatocyte–HSC signaling, hepatocyte–LSEC-mediated vascular remodeling, and the emergence of exosomal miRNA exchange collectively drive the transition from activated fibrogenic stroma to CAF-dominated support of premalignant hepatocytes [5,8,15,40,181]. Once established, CAFs in turn promote oncogenesis by secreting MMPs, TGF-β, VEGF, chemokines, and exosomal miRNAs such as miR-21 that enhance epithelial–mesenchymal plasticity, invasion, angiogenesis, and immune evasion, thereby reinforcing the transformation of non-tumor hepatocytes and surrounding tissue into a tumor-permissive microenvironment [8,9,52,53,194,203,204,206].

### 5.5. Immune Suppression at the PME–TME Interface

In advanced HBV-induced fibrosis, miRNAs regulate gene expression in a microenvironment that becomes progressively populated by dysfunctional immune cell types that promote its advance to the PME. Ongoing HBV antigen and cytokine hepatocyte exposure eventually promotes T-cell exhaustion, reduced cytotoxic activity, and an increase in PD-1/PD-L1-associated inhibition [13,24,189,190]. In parallel, dysfunctional NK cells, Tregs, MDSCs, and TAMs become increasingly dominant, secreting IL-10, TGF-β, VEGF, and chemokines that further suppress antiviral and antitumor immunity while continuing to promote HSC activation, angiogenesis, epithelial plasticity, and CAF persistence [8,13,24,189,190]. Examples of miRNA regulating this microenvironment include miR-223, which regulates PDGF-induced HSC activation by targeting NLRP3/FOXO3-associated inflammatory pathways to influence ongoing innate immune response [108,109]. Other miRNAs like upregulated miR-21 and miR-221/222 also regulate pro-survival, fibrogenic, and oncogenic signaling in hepatocytes and stromal cells, while the CAF-induced exosomal miR-20a-5p and downregulated miR-150-3p promote Wnt/β-catenin- and RAS/MAPK-associated migration, invasion, and epithelial–mesenchymal plasticity [31,36,52,53,178]. Many of these TAM- and CAF-induced mechanisms have only been experimentally validated in advanced HCC rather than in HBV premalignant tissue. However, this evidence promotes a hypothesis that miRNA regulation of the HBV-associated PME facilitates the cooperation between a tolerogenic microenvironment that facilitates ECM stiffness, hypoxia, angiogenesis, and CAF activation to promote early HBV-HCC [13,201,202,203].

## 6. Conclusions and Implications for Diagnosis and Therapy

This review highlights that HBV-HCC pathogenesis is the consequence of multicellular dysregulation rather than solely the accumulation of mutations in infected hepatocytes. The PME/TME transition arises from a staged, miRNA-driven remodeling of the hepatic microenvironment, beginning with antiviral signaling in HBV-infected hepatocytes and progressing through HSC activation, ECM remodeling, mechanotransduction, angiogenesis, and CAF emergence. Across this continuum, selected dysregulated miRNAs orchestrate hepatocyte-intrinsic antiviral pathways and stromal responses so that HBV-HCC can be viewed as a sequence of interconnected, miRNA-regulated hepatocyte–stromal states rather than a simple transition from CHB to overt cancer.

We propose that miRNA signatures at these defined stages could be evaluated as early risk biomarkers for hepatocyte injury, HSC activation, angiogenesis, mechanotransduction, and CAF programing. Dysregulated circulating or exosomal miRNAs such as miR-21, miR-29a/b, miR-122, miR-146a, miR-155, miR-200a, miR-126, miR-210, and members of the miR-130/301 family can be mechanistically linked to specific phases of HBV-HCC pathogenesis before a tumor microenvironment is fully established. Integrated, stage-specific miRNA panels, combined with histology and clinical outcomes, therefore offer the possibility of increased diagnostic precision and improved risk stratification for CHB patients.

The same miRNA networks also provide a framework for therapeutic intervention that targets the liver microenvironment rather than hepatocytes in isolation. Antimirs directed against upregulated pro-fibrotic and pro-tumorigenic miRNAs (for example, miR-21, miR-17-5p, miR-27a, miR-33a, miR-130/301 and miR-221/222), together with mimics of downregulated tumor-suppressive miRNAs (such as miR-29a/b, miR-122, miR-200 family members, and miR-126), could hypothetically attenuate HSC activation, ECM deposition and stiffness, angiogenesis, and CAF programing. Because miRNAs regulate multiple targets in NF-κB, interferon, TGF-β/SMAD, Wnt/β-catenin, Hippo/YAP, and VEGF pathways, carefully designed miRNA-based interventions might modulate several dysregulated signaling axes simultaneously and could be explored as adjuvant therapies alongside conventional antiviral and anti-fibrotic treatments.

Realizing this potential will require cell-specific and stage-resolved approaches that integrate miRNA biology with modern profiling and model systems. The translation of miRNA-based therapeutics has been confronted by a range of fundamental biological and computational barriers that are yet to be overcome. These barriers include issues related to cancer drug resistance like intrinsic resistance, tumor microenvironment heterogeneity, controlling their multi-target evolutionary design, and managing off-target and immune-related events. It is anticipated, however, that emerging AI and multi-omics technologies could solve some of these fundamental biological issues, especially the interpretation of dynamic heterogenous datasets. The design of miRNA-based diagnostics and therapeutics should incorporate single-cell and spatial transcriptomic profiling, organoid and co-culture systems, and in-vivo models that allow selective delivery to hepatocytes, HSCs, LSECs, or CAFs, thereby minimizing off-target effects. Integrating mechanistically validated miRNA biomarkers with microenvironment-targeted miRNA drugs may ultimately offer an opportunity to intercept HBV-HCC development and to reverse key pathogenic features of HBV-induced liver disease before a CAF-rich tumor microenvironment becomes irreversible.

## Figures and Tables

**Table 2:** Key miRNAs regulating fibrogenesis.

MiRNA	HBV	Target	Regulation of Fibrogenesis	Reference
mir-17-5p	Down	WIF1/SMAD7	Activates HSCs represses WIF1/SMAD7	[32,151,152]
miR-21-5p	Down	SMAD7/PTEN	Promotes SMAD2/3/4/TGF-B expression	[30,138,153,154]
miR-27a	Up	PPARy/FOXO1/APC/p53	Promotes upregulation of COL1A1,	[33]
miR-33a	Up	SMAD7/PPAR α	Promotes SMAD 2/4 and aHSC	[34,129,155]
miR-132	Up	SIRT1/CTGF	Represses COL1A1/TIMP3/CTGF	[156,157,158]
miR-181b	Up	p27/PTEN	Promotes HSC/MMP/α-SMA expression	[35,137,159]
miR-214-5p	Up	CTGF/TGF-B	Represses aHSC by HSC apoptosis	[160,161]
miR-222	Up	TFRC	Reduces ferroptosis regulation of HSCs	[36,116,162]
miR-942	Up	BAMBI	Represses BAMB1 to activate HSCs	[155,163]
miR-18a	Down	CTGF	Fails to regulate HSC activation	[82,155]
miR-19b	Down	TGF-B/GRB2	Reduced inhibition TGF-B/aHSC/COL1A1	[127]
miR-185	Down	RICTOR/RHEB/SREBF1	Activates HSCs by failing to regulate	[155,164,165]
miR-23a-5p	Up	ERK/BMP2/CAV-1/CDH1	Induces apoptosis to repress HSCs	[166]
miR-23b/-27b	Up	LOX/CTGF/TIMP1/Grem1	Fails to repress ECM, TIMP1 and aHSC	[167,168]
miR-29b	Down	TGF-B/P13K/AKT	Fails to regulate COL1A1/TIMP-1/α-SMA,	[27,28,105]
miR-29a	Down	IGF-1/PDGF	Fails to repress collagen 1, 3, 4/ECM	[29,105,153]
miR-30	Up	SNAIL1	Fails to regulate SNAIL1 led EMT/ECM	[169]
miR-30e	Down	P4HA2	Enhances collagen depositions	[83]
miR-34a-5p	Up	NF-kB/HMGB1/SMAD4	HMGB1 activation > JNK/ERK autophagy	[47,155]
miR-101b	Down	TGF-B/P13K/AKT/mTOR	Fails to repress α-SMA, collagen 1	[139,140]
miR-122	Down	TGFB1/KLF6/P4HA1	Fails to repress HSC collagen, α-SMA	[135,170]
miR-146a	Up	Il-6/SMAD4	Fails to repress IL-6 induced aHSC	[171,172]
miR-148a	Up	IkB-α/ NUMB/ NOTCH	Fails to repress NOTCH signaling	[173]
miR-150	Down	c-myb/Rac-1	Fails to regulate α-SMA/COL1A1	[174]
miR-152/-378a	Down	Gli3	Fails to repress collagen, α-SMA	[175,176]
miR-200a	Up	B-CAT	Fails to repress HSC activation	[49]

## Data Availability

No new data were created or analyzed in this study. Data sharing is not applicable to this article.

## Supplementary Materials

The following supporting information can be downloaded at: https://www.mdpi.com/article/10.3390/ijms27177581/s1.

## References

[1] Al-Busafi S.A., Alwassief A. (2024). Global perspectives on the hepatitis B vaccination: Challenges, achievements, and the road to elimination by 2030. Vaccines.

[2] Lok A.S., McMahon B.J. (2007). Chronic hepatitis B. Hepatology.

[3] Ibrahim S., El-Din S., Bazzal I. (2006). Antibody level after hepatitis-B vaccination in hemodialysis patients: Impact of dialysis adequacy, chronic inflammation, local endemicity and nutritional status. J. Natl. Med. Assoc..

[4] Stanaway J.D., Flaxman A.D., Naghavi M., Fitzmaurice C., Vos T., Abubakar I., Abu-Raddad L.J., Assadi R., Bhala N., Cowie B. (2016). The global burden of viral hepatitis from 1990 to 2013: Findings from the Global Burden of Disease Study 2013. Lancet.

[5] Koyama Y., Brenner D.A. (2017). Liver inflammation and fibrosis. J. Clin. Investig..

[6] Hammerich L., Tacke F. (2023). Hepatic inflammatory responses in liver fibrosis. Nat. Rev. Gastroenterol. Hepatol..

[7] Arzumanyan A., Reis H.M., Feitelson M.A. (2013). Pathogenic mechanisms in HBV-and HCV-associated hepatocellular carcinoma. Nat. Rev. Cancer.

[8] Affo S., Yu L.-X., Schwabe R.F. (2017). The role of cancer-associated fibroblasts and fibrosis in liver cancer. Annu. Rev. Pathol. Mech. Dis..

[9] Zhao Y., Shen M., Wu L., Yang H., Yao Y., Yang Q., Du J., Liu L., Li Y., Bai Y. (2023). Stromal cells in the tumor microenvironment: Accomplices of tumor progression?. Cell Death Dis..

[10] Ringelhan M., O’connor T., Protzer U., Heikenwalder M. (2015). The direct and indirect roles of HBV in liver cancer: Prospective markers for HCC screening and potential therapeutic targets. J. Pathol..

[11] Yang T., Gu Z., Feng J., Shan J., Qian C., Zhuang N. (2025). Non-parenchymal cells: Key targets for modulating chronic liver diseases. Front. Immunol..

[12] Lin J., Gehring A.J. (2025). Role of Immune Cells in Hepatitis B Virus and Associated Sequelae. Annu. Rev. Pathol. Mech. Dis..

[13] Iannacone M., Guidotti L.G. (2022). Immunobiology and pathogenesis of hepatitis B virus infection. Nat. Rev. Immunol..

[14] Ringelhan M., McKeating J.A., Protzer U. (2017). Viral hepatitis and liver cancer. Philos. Trans. R. Soc. Lond. B Biol. Sci..

[15] Szabo G., Bala S. (2013). MicroRNAs in liver disease. Nat. Rev. Gastroenterol. Hepatol..

[16] Sartorius K., Makarova J., Sartorius B., An P., Winkler C., Chuturgoon A., Kramvis A. (2019). The regulatory role of microRNA in hepatitis-B virus-associated hepatocellular carcinoma (HBV-HCC) pathogenesis. Cells.

[17] Hou J., Wang P., Lin L., Liu X., Ma F., An H., Wang Z., Cao X. (2009). MicroRNA-146a feedback inhibits RIG-I-dependent Type I IFN production in macrophages by targeting TRAF6, IRAK1, and IRAK2. J. Immunol..

[18] Xu D., Han Q., Hou Z., Zhang C., Zhang J. (2017). MiR-146a negatively regulates NK cell functions via STAT1 signaling. Cell. Mol. Immunol..

[19] Su C., Hou Z., Zhang C., Tian Z., Zhang J. (2011). Ectopic expression of microRNA-155 enhances innate antiviral immunity against HBV infection in human hepatoma cells. Virol. J..

[20] Wang S., Qiu L., Yan X., Jin W., Wang Y., Chen L., Wu E., Ye X., Gao G.F., Wang F. (2012). Loss of microRNA 122 expression in patients with hepatitis B enhances hepatitis B virus replication through cyclin G1-modulated P53 activity. Hepatology.

[21] Xu H., Xu S.-J., Xie S.-J., Zhang Y., Yang J.-H., Zhang W.-Q., Zheng M.-N., Zhou H., Qu L.-H. (2019). MicroRNA-122 supports robust innate immunity in hepatocytes by targeting the RTKs/STAT3 signaling pathway. eLife.

[22] Fu L., Fu X., Mo J., Li X., Li R., Peng S. (2019). MiR-146a-5p enhances hepatitis B virus replication through autophagy to promote aggravation of chronic hepatitis B. IUBMB Life.

[23] Liu Y., Qin L., Wang J., Xie X., Zhang Y., Li C., Guan Z., Qian L., Chen L., Hu J. (2022). MiR-146a maintains immune tolerance of Kupffer cells and facilitates hepatitis B virus persistence in mice. J. Immunol..

[24] Eichmüller S.B., Osen W., Mandelboim O., Seliger B. (2017). Immune modulatory microRNAs involved in tumor attack and tumor immune escape. JNCI J. Natl. Cancer Inst..

[25] Wieland S.F., Chisari F.V. (2005). Stealth and cunning: Hepatitis B and hepatitis C viruses. J. Virol..

[26] Mederacke I., Hsu C.C., Tröger J., Huebener P., Dapito D.H., Jean-Philippe P., Schwabe R. (2013). Hepatic Stellate Cells Are the Dominant Source of Myofibroblasts Across all Types of Chronic Liver Injury but do not Contribute to the Generation of Hepatocytes: 60. Hepatology.

[27] Roderburg C., Urban G.W., Bettermann K., Vucur M., Zimmermann H., Schmidt S., Janssen J., Koppe C., Knolle P., Castoldi M. (2011). Micro-RNA profiling reveals a role for miR-29 in human and murine liver fibrosis. Hepatology.

[28] Wang J., Chu E.S., Chen H.-Y., Man K., Go M.Y., Huang X.R., Lan H.Y., Sung J.J., Yu J. (2014). MicroRNA-29b prevents liver fibrosis by attenuating hepatic stellate cell activation and inducing apoptosis through targeting PI3K/AKT pathway. Oncotarget.

[29] Kwiecinski M., Elfimova N., Noetel A., Töx U., Steffen H.-M., Hacker U., Nischt R., Dienes H.P., Odenthal M. (2012). Expression of platelet-derived growth factor-C and insulin-like growth factor I in hepatic stellate cells is inhibited by miR-29. Lab. Investig..

[30] Zhang Z., Zha Y., Hu W., Huang Z., Gao Z., Zang Y., Chen J., Dong L., Zhang J. (2013). The autoregulatory feedback loop of microRNA-21/programmed cell death protein 4/activation protein-1 (MiR-21/PDCD4/AP-1) as a driving force for hepatic fibrosis development. J. Biol. Chem..

[31] Li W., Yu X., Chen X., Wang Z., Yin M., Zhao Z., Zhu C. (2021). HBV induces liver fibrosis via the TGF-β1/miR-21-5p pathway. Exp. Ther. Med..

[32] Yu F., Lu Z., Huang K., Wang X., Xu Z., Chen B., Dong P., Zheng J. (2015). MicroRNA-17-5p-activated Wnt/β-catenin pathway contributes to the progression of liver fibrosis. Oncotarget.

[33] Zhang H., Yan X.-L., Guo X.-X., Shi M.-J., Lu Y.-Y., Zhou Q.-M., Chen Q.-L., Hu Y.-Y., Xu L.-M., Huang S. (2018). MiR-27a as a predictor for the activation of hepatic stellate cells and hepatitis B virus-induced liver cirrhosis. Oncotarget.

[34] Li Z.-J., Ou-Yang P.-H., Han X.-P. (2014). Profibrotic effect of miR-33a with Akt activation in hepatic stellate cells. Cell. Signal..

[35] Zheng J., Wu C., Xu Z., Xia P., Dong P., Chen B., Yu F. (2015). Hepatic stellate cell is activated by microRNA-181b via PTEN/Akt pathway. Mol. Cell. Biochem..

[36] Ogawa T., Enomoto M., Fujii H., Sekiya Y., Yoshizato K., Ikeda K., Kawada N. (2012). MicroRNA-221/222 upregulation indicates the activation of stellate cells and the progression of liver fibrosis. Gut.

[37] Fu M., Yin W., Zhang W., Zhu Y., Ni H., Gong L. (2022). MicroRNA-15a inhibits hepatic stellate cell activation and proliferation via targeting SRY-box transcription factor 9. Bioengineered.

[38] Kisseleva T., Brenner D. (2021). Molecular and cellular mechanisms of liver fibrosis and its regression. Nat. Rev. Gastroenterol. Hepatol..

[39] Friedman S.L. (2008). Hepatic stellate cells: Protean, multifunctional, and enigmatic cells of the liver. Physiol. Rev..

[40] Tsuchida T., Friedman S.L. (2017). Mechanisms of hepatic stellate cell activation. Nat. Rev. Gastroenterol. Hepatol..

[41] Fish J.E., Santoro M.M., Morton S.U., Yu S., Yeh R.-F., Wythe J.D., Ivey K.N., Bruneau B.G., Stainier D.Y., Srivastava D. (2008). MiR-126 regulates angiogenic signaling and vascular integrity. Dev. Cell.

[42] Fasanaro P., D’Alessandra Y., Di Stefano V., Melchionna R., Romani S., Pompilio G., Capogrossi M.C., Martelli F. (2008). MicroRNA-210 modulates endothelial cell response to hypoxia and inhibits the receptor tyrosine kinase ligand Ephrin-A3. J. Biol. Chem..

[43] Wang S., Aurora A.B., Johnson B.A., Qi X., McAnally J., Hill J.A., Richardson J.A., Bassel-Duby R., Olson E.N. (2008). The endothelial-specific microRNA miR-126 governs vascular integrity and angiogenesis. Dev. Cell.

[44] Bertero T., Cottrill K.A., Annis S., Bhat B., Gochuico B.R., Osorio J.C., Rosas I., Haley K.J., Corey K.E., Chung R.T. (2015). A YAP/TAZ-miR-130/301 molecular circuit exerts systems-level control of fibrosis in a network of human diseases and physiologic conditions. Sci. Rep..

[45] Bertero T., Cottrill K.A., Lu Y., Haeger C.M., Dieffenbach P., Annis S., Hale A., Bhat B., Kaimal V., Zhang Y.-Y. (2015). Matrix remodeling promotes pulmonary hypertension through feedback mechanoactivation of the YAP/TAZ-miR-130/301 circuit. Cell Rep..

[46] Shen S., Guo X., Yan H., Lu Y., Ji X., Li L., Liang T., Zhou D., Feng X.-H., Zhao J.C. (2015). A miR-130a-YAP positive feedback loop promotes organ size and tumorigenesis. Cell Res..

[47] Feili X., Wu S., Ye W., Tu J., Lou L. (2018). MicroRNA-34a-5p inhibits liver fibrosis by regulating TGF-β1/Smad3 pathway in hepatic stellate cells. Cell Biol. Int..

[48] Zhang J., Wang H., Yao L., Zhao P., Wu X. (2021). MiR-34a promotes fibrosis of hepatic stellate cells via the TGF-β pathway. Ann. Transl. Med..

[49] Sun X., He Y., Ma T.-T., Huang C., Zhang L., Li J. (2014). Participation of miR-200a in TGF-β1-mediated hepatic stellate cell activation. Mol. Cell. Biochem..

[50] Xia H., Cheung W.K., Sze J., Lu G., Jiang S., Yao H., Bian X.-W., Poon W.S., Kung H.-F., Lin M.C. (2010). MiR-200a regulates epithelial-mesenchymal to stem-like transition via ZEB2 and β-catenin signaling. J. Biol. Chem..

[51] Li X., Zhang W., Xu K., Lu J. (2020). MiR-34a promotes liver fibrosis in patients with chronic hepatitis via mediating Sirt1/p53 signaling pathway. Pathol.-Res. Pract..

[52] Qi Y., Wang H., Zhang Q., Liu Z., Wang T., Wu Z., Wu W. (2022). CAF-released exosomal miR-20a-5p facilitates HCC progression via the LIMA1-mediated β-catenin pathway. Cells.

[53] Yugawa K., Yoshizumi T., Mano Y., Itoh S., Harada N., Ikegami T., Kohashi K., Oda Y., Mori M. (2021). Cancer-associated fibroblasts promote hepatocellular carcinoma progression through downregulation of exosomal miR-150-3p. Eur. J. Surg. Oncol..

[54] Ma S., Qin K., Ouyang H., Zhu H., Lei P., Shen G. (2020). HBV infection exacerbates PTEN defects in hepatocellular carcinoma through upregulation of miR-181a/382/362/19a. Am. J. Transl. Res..

[55] Matsuzaki J., Suzuki H. (2015). Role of MicroRNAs-221/222 in digestive systems. J. Clin. Med..

[56] Song Q., An Q., Niu B., Lu X., Zhang N., Cao X. (2019). Role of miR-221/222 in tumor development and the underlying mechanism. J. Oncol..

[57] Wang Y., Lee C.G. (2011). Role of miR-224 in hepatocellular carcinoma: A tool for possible therapeutic intervention?. Epigenomics.

[58] Liu P., Xia P., Fu Q., Liu C., Luo Q., Cheng L., Yu P., Qin T., Zhang H. (2021). MiR-199a-5p inhibits the proliferation of hepatocellular carcinoma cells by regulating CDC25A to induce cell cycle arrest. Biochem. Biophys. Res. Commun..

[59] Ghosh A., Dasgupta D., Ghosh A., Roychoudhury S., Kumar D., Gorain M., Butti R., Datta S., Agarwal S., Gupta S. (2017). MiRNA199a-3p suppresses tumor growth, migration, invasion and angiogenesis in hepatocellular carcinoma by targeting VEGFA, VEGFR1, VEGFR2, HGF and MMP2. Cell Death Dis..

[60] Xu J., An P., Winkler C.A., Yu Y. (2020). Dysregulated microRNAs in hepatitis B virus-related hepatocellular carcinoma: Potential as biomarkers and therapeutic targets. Front. Oncol..

[61] Mannaerts I., Leite S.B., Verhulst S., Claerhout S., Eysackers N., Thoen L.F., Hoorens A., Reynaert H., Halder G., van Grunsven L.A. (2015). The Hippo pathway effector YAP controls mouse hepatic stellate cell activation. J. Hepatol..

[62] Dupont S., Morsut L., Aragona M., Enzo E., Giulitti S., Cordenonsi M., Zanconato F., Le Digabel J., Forcato M., Bicciato S. (2011). Role of YAP/TAZ in mechanotransduction. Nature.

[63] Robek M.D., Boyd B.S., Wieland S.F., Chisari F.V. (2004). Signal transduction pathways that inhibit hepatitis B virus replication. Proc. Natl. Acad. Sci. USA.

[64] Li Q., Sun B., Zhuo Y., Jiang Z., Li R., Lin C., Jin Y., Gao Y., Wang D. (2022). Interferon and interferon-stimulated genes in HBV treatment. Front. Immunol..

[65] Sun B., Karin M. (2008). NF-κB signaling, liver disease and hepatoprotective agents. Oncogene.

[66] Halemubieke S., Liu X., Yan Y., Ji H., Sun H., Ma J., Chang L., Wang L. (2026). New Insights into the Role of NF-κB in Hepatitis B Virus Infection. Int. J. Biol. Sci..

[67] Sato S., Li K., Kameyama T., Hayashi T., Ishida Y., Murakami S., Watanabe T., Iijima S., Sakurai Y., Watashi K. (2015). The RNA sensor RIG-I dually functions as an innate sensor and direct antiviral factor for hepatitis B virus. Immunity.

[68] Chen Q., Sun L., Chen Z.J. (2016). Regulation and function of the cGAS–STING pathway of cytosolic DNA sensing. Nat. Immunol..

[69] Hou Z., Zhang J., Han Q., Su C., Qu J., Xu D., Zhang C., Tian Z. (2016). Hepatitis B virus inhibits intrinsic RIG-I and RIG-G immune signaling via inducing miR146a. Sci. Rep..

[70] Xu Z.-Y., Gao J.-S., He Y., Xiao X.-Q., Gong G.-Z., Zhang M. (2025). Hepatitis B virus confers innate immunity evasion through hepatitis B virus-miR-3 down-regulation of cGAS-Sting-IFN signaling. World J. Hepatol..

[71] Momeni M., Hassanshahi G., Arababadi M.K., Kennedy D. (2014). Ectopic expression of micro-RNA-1, 21 and 125a in peripheral blood immune cells is associated with chronic HBV infection. Mol. Biol. Rep..

[72] Yu F., Zhou G., Li G., Chen B., Dong P., Zheng J. (2015). Serum miR-181b is correlated with hepatitis B virus replication and disease progression in chronic hepatitis B patients. Dig. Dis. Sci..

[73] Zhao L., Yuan H., Wang Y., Geng Y., Yun H., Zheng W., Yuan Y., Lv P., Hou C., Zhang H. (2023). HBV confers innate immune evasion through triggering HAT1/acetylation of H4K5/H4K12/miR-181a-5p or KPNA2/cGAS-STING/IFN-I signaling. J. Med. Virol..

[74] Li J.-F., Dai X.-P., Zhang W., Sun S.-H., Zeng Y., Zhao G.-Y., Kou Z.-H., Guo Y., Yu H., Du L.-Y. (2015). Upregulation of microRNA-146a by hepatitis B virus X protein contributes to hepatitis development by downregulating complement factor H. MBio.

[75] Wang W., Bian H., Li F., Li X., Zhang D., Sun S., Song S., Zhu Q., Ren W., Qin C. (2018). HBeAg induces the expression of macrophage miR-155 to accelerate liver injury via promoting production of inflammatory cytokines. Cell. Mol. Life Sci..

[76] Zhang T., Yang Z., Kusumanchi P., Han S., Liangpunsakul S. (2020). Critical role of microRNA-21 in the pathogenesis of liver diseases. Front. Med..

[77] Song K., Han C., Zhang J., Lu D., Dash S., Feitelson M., Lim K., Wu T. (2013). Epigenetic regulation of miR-122 by PPARγ and hepatitis B virus X protein in hepatocellular carcinoma cells. Hepatology.

[78] Sartorius K., An P., Winkler C., Chuturgoon A., Li X., Makarova J., Kramvis A. (2021). The epigenetic modulation of cancer and immune pathways in hepatitis B virus-associated hepatocellular carcinoma: The influence of HBx and miRNA dysregulation. Front. Immunol..

[79] Sartorius K., Swadling L., An P., Makarova J., Winkler C., Chuturgoon A., Kramvis A. (2020). The multiple roles of hepatitis B virus X protein (HBx) dysregulated microRNA in hepatitis B virus-associated hepatocellular carcinoma (HBV-HCC) and immune pathways. Viruses.

[80] Damania P., Sen B., Dar S.B., Kumar S., Kumari A., Gupta E., Sarin S.K., Venugopal S.K. (2014). Hepatitis B virus induces cell proliferation via HBx-induced microRNA-21 in hepatocellular carcinoma by targeting programmed cell death protein4 (PDCD4) and phosphatase and tensin homologue (PTEN). PLoS ONE.

[81] Khosravi M., Behboudi E., Razavi-Nikoo H., Tabarraei A. (2024). Hepatitis B virus X protein induces expression changes of miR-21, miR-22, miR-122, miR-132, and miR-222 in Huh-7 cell line. Arch. Razi Inst..

[82] Liu X., Zhang Y., Wang P., Wang H., Su H., Zhou X., Zhang L. (2016). HBX protein-induced downregulation of microRNA-18a is responsible for upregulation of connective tissue growth factor in HBV infection-associated hepatocarcinoma. Med. Sci. Monit. Int. Med. J. Exp. Clin. Res..

[83] Feng G., Li J., Yang Z., Zhang S., Liu Y., Zhang W., Ye L., Zhang X. (2017). Hepatitis B virus X protein promotes the development of liver fibrosis and hepatoma through downregulation of miR-30e targeting P4HA2 mRNA. Oncogene.

[84] Singh A.K., Rooge S.B., Varshney A., Vasudevan M., Bhardwaj A., Venugopal S.K., Trehanpati N., Kumar M., Geffers R., Kumar V. (2018). Global microRNA expression profiling in the liver biopsies of hepatitis B virus–infected patients suggests specific microRNA signatures for viral persistence and hepatocellular injury. Hepatology.

[85] Deng W., Zhang X., Ma Z., Lin Y., Lu M. (2017). MicroRNA-125b-5p mediates post-transcriptional regulation of hepatitis B virus replication via the LIN28B/let-7 axis. RNA Biol..

[86] Ge J., Huang Z., Liu H., Chen J., Xie Z., Chen Z., Peng J., Sun J., Hou J., Zhang X. (2017). Lower Expression of MicroRNA-155 Contributes to Dysfunction of Natural Killer Cells in Patients with Chronic Hepatitis B. Front. Immunol..

[87] Li G.Y., Zhou Y., Ying R.S., Shi L., Cheng Y.Q., Ren J.P., Griffin J.W., Jia Z.S., Li C.F., Moorman J.P. (2015). Hepatitis C virus–induced reduction in miR-181a impairs CD4+ T-cell responses through overexpression of DUSP6. Hepatology.

[88] Zou C., Chen J., Chen K., Wang S., Cao Y., Zhang J., Sheng Y., Huang A., Tang H. (2015). Functional analysis of miR-181a and Fas involved in hepatitis B virus-related hepatocellular carcinoma pathogenesis. Exp. Cell Res..

[89] Ostrycharz E., Hukowska-Szematowicz B. (2022). Micro-players of great significance—Host microRNA signature in viral infections in humans and animals. Int. J. Mol. Sci..

[90] Ko C., Lee S., Windisch M.P., Ryu W.-S. (2014). DDX3 DEAD-box RNA helicase is a host factor that restricts hepatitis B virus replication at the transcriptional level. J. Virol..

[91] Wendlandt E.B., Graff J.W., Gioannini T.L., McCaffrey A.P., Wilson M.E. (2012). The role of microRNAs miR-200b and miR-200c in TLR4 signaling and NF-κB activation. Innate Immun..

[92] Tian H., He Z. (2018). MiR-200c targets nuclear factor IA to suppress HBV replication and gene expression via repressing HBV Enhancer I activity. Biomed. Pharmacother..

[93] Qiu X., Dong S., Qiao F., Lu S., Song Y., Lao Y., Li Y., Zeng T., Hu J., Zhang L. (2013). HBx-mediated miR-21 upregulation represses tumor-suppressor function of PDCD4 in hepatocellular carcinoma. Oncogene.

[94] Chen Y., Chen J., Wang H., Shi J., Wu K., Liu S., Liu Y., Wu J. (2013). HCV-induced miR-21 contributes to evasion of host immune system by targeting MyD88 and IRAK1. PLoS Pathog..

[95] Zhao Y., Yu Y., Ye L. (2019). MiR-3613-3p impairs IFN-induced immune response by targeting CMPK1 in chronic hepatitis B. Infect. Genet. Evol..

[96] Zhao X., Sun L., Mu T., Yi J., Ma C., Xie H., Liu M., Tang H. (2020). An HBV-encoded miRNA activates innate immunity to restrict HBV replication. J. Mol. Cell Biol..

[97] Song K., Han C., Dash S., Balart L.A., Wu T. (2015). MiR-122 in hepatitis B virus and hepatitis C virus dual infection. World J. Hepatol..

[98] Hong Z., Hong H., Liu J., Zheng X., Huang M., Li C., Xia J. (2015). MiR-106a is downregulated in peripheral blood mononuclear cells of chronic hepatitis B and associated with enhanced levels of interleukin-8. Mediat. Inflamm..

[99] Bannazadeh Baghi H., Bayat M., Mehrasa P., Alavi S.M.A., Lotfalizadeh M.H., Memar M.Y., Taghavi S.P., Zarepour F., Hamblin M.R., Sadri Nahand J. (2024). Regulatory role of microRNAs in virus-mediated inflammation. J. Inflamm..

[100] Mahe Y., Mukaida N., Kuno K., Akiyama M., Ikeda N., Matsushima K., Murakami S. (1991). Hepatitis B virus X protein transactivates human interleukin-8 gene through acting on nuclear factor kB and CCAAT/enhancer-binding protein-like cis-elements. J. Biol. Chem..

[101] Li Y., Xie J., Xu X., Wang J., Ao F., Wan Y., Zhu Y. (2013). MicroRNA-548 down-regulates host antiviral response via direct targeting of IFN-λ1. Protein Cell.

[102] Kitab B., Alj H.S., Ezzikouri S., Benjelloun S. (2015). MicroRNAs as important players in host-hepatitis B virus interactions. J. Clin. Transl. Hepatol..

[103] Fang J., Zhuge L., Rao H., Huang S., Jin L., Li J. (2019). Increased levels of miR-155 are related to higher T-cell activation in the peripheral blood of patients with chronic hepatitis B. Genet. Test. Mol. Biomark..

[104] Yao X.-C., Wu J.-J., Yuan S.-T., Yuan F.-L. (2025). Recent insights and perspectives into the role of the miRNA-29 family in innate immunity. Int. J. Mol. Med..

[105] Huang C., Zheng J.M., Cheng Q., Yu K.K., Ling Q.X., Chen M.Q., Li N. (2014). Serum micro RNA-29 levels correlate with disease progression in patients with chronic hepatitis B virus infection. J. Dig. Dis..

[106] Xing T., Jiang D., Huang J., Xu Z. (2014). Expression and clinical significance of miR-122 and miR-29 in hepatitis B virus-related liver. Genet. Mol. Res..

[107] Novellino L., Rossi R.L., Bonino F., Cavallone D., Abrignani S., Pagani M., Brunetto M.R. (2012). Circulating hepatitis B surface antigen particles carry hepatocellular microRNAs. PLoS ONE.

[108] Ye D., Zhang T., Lou G., Liu Y. (2018). Role of miR-223 in the pathophysiology of liver diseases. Exp. Mol. Med..

[109] Chen L., Song Y., He L., Wan X., Lai L., Dai F., Liu Y., Wang Q. (2016). MicroRNA-223 promotes type I interferon production in antiviral innate immunity by targeting forkhead box protein O_3_ (FOXO_3_). J. Biol. Chem..

[110] Taganov K.D., Boldin M.P., Chang K.-J., Baltimore D. (2006). NF-κB-dependent induction of microRNA miR-146, an inhibitor targeted to signaling proteins of innate immune responses. Proc. Natl. Acad. Sci. USA.

[111] Mann M., Mehta A., Zhao J.L., Lee K., Marinov G.K., Garcia-Flores Y., Lu L.-F., Rudensky A.Y., Baltimore D. (2017). An NF-κB-microRNA regulatory network tunes macrophage inflammatory responses. Nat. Commun..

[112] O’Connell R.M., Rao D.S., Baltimore D. (2012). MicroRNA regulation of inflammatory responses. Annu. Rev. Immunol..

[113] Hou Z.H., Han Q.J., Zhang C., Tian Z.G., Zhang J. (2014). MiR146a impairs the IFN-induced anti-HBV immune response by downregulating STAT1 in hepatocytes. Liver Int..

[114] Ezeonwumelu I.J., Garcia-Vidal E., Ballana E. (2021). JAK-STAT pathway: A novel target to tackle viral infections. Viruses.

[115] Yu Y., Dong H., Zhang Y., Sun J., Li B., Chen Y., Feng M., Yang X., Gao S., Jiang W. (2022). MicroRNA-223 downregulation promotes HBx-induced podocyte pyroptosis by targeting the NLRP3 inflammasome: Y. Yu et al. Arch. Virol..

[116] Zhang Q., Qu Y., Zhang Q., Li F., Li B., Li Z., Dong Y., Lu L., Cai X. (2023). Exosomes derived from hepatitis B virus-infected hepatocytes promote liver fibrosis via miR-222/TFRC axis. Cell Biol. Toxicol..

[117] Shi Y., Du L., Lv D., Li Y., Zhang Z., Huang X., Tang H. (2021). Emerging role and therapeutic application of exosome in hepatitis virus infection and associated diseases. J. Gastroenterol..

[118] Bertoletti A., Ferrari C. (2016). Adaptive immunity in HBV infection. J. Hepatol..

[119] Bertoletti A., Gehring A.J. (2006). The immune response during hepatitis B virus infection. J. General. Virol..

[120] Zhou R., O’hara S.P., Chen X.-M. (2011). MicroRNA regulation of innate immune responses in epithelial cells. Cell. Mol. Immunol..

[121] Chen Y., Shen A., Rider P.J., Yu Y., Wu K., Mu Y., Hao Q., Liu Y., Gong H., Zhu Y. (2011). A liver-specific microRNA binds to a highly conserved RNA sequence of hepatitis B virus and negatively regulates viral gene expression and replication. FASEB J..

[122] Xing T., Xu H., Yu W., Wang B., Zhang J. (2015). Expression profile and clinical significance of miRNAs at different stages of chronic hepatitis B virus infection. Int. J. Clin. Exp. Med..

[123] Wang S., Zhang X., Ju Y., Zhao B., Yan X., Hu J., Shi L., Yang L., Ma Z., Chen L. (2013). MicroRNA-146a feedback suppresses T cell immune function by targeting Stat1 in patients with chronic hepatitis B. J. Immunol..

[124] Nakamura M., Kanda T., Jiang X., Haga Y., Takahashi K., Wu S., Yasui S., Nakamoto S., Yokosuka O. (2017). Serum microRNA-122 and Wisteria floribunda agglutinin-positive Mac-2 binding protein are useful tools for liquid biopsy of the patients with hepatitis B virus and advanced liver fibrosis. PLoS ONE.

[125] Roehlen N., Crouchet E., Baumert T.F. (2020). Liver fibrosis: Mechanistic concepts and therapeutic perspectives. Cells.

[126] Bataller R., Brenner D.A. (2005). Liver fibrosis. J. Clin. Investig..

[127] Lakner A.M., Steuerwald N.M., Walling T.L., Ghosh S., Li T., McKillop I.H., Russo M.W., Bonkovsky H.L., Schrum L.W. (2012). Inhibitory effects of microRNA 19b in hepatic stellate cell-mediated fibrogenesis. Hepatology.

[128] Liao X., Liu R., Zhou T., Zhao X., Xiong X., Zhang K., Zheng Z., Xia X., Tang C., Gong Y. (2025). MiRNA-29b accelerates the PDGF in exosomes and stimulates hepatic stellate cells to promote liver fibrosis in biliary atresia. Eur. J. Med. Res..

[129] Huang C.-F., Sun C.-C., Zhao F., Zhang Y.-D., Li D.-J. (2015). MiR-33a levels in hepatic and serum after chronic HBV-induced fibrosis. J. Gastroenterol..

[130] Rao S., Zaidi S., Banerjee J., Jogunoori W., Sebastian R., Mishra B., Nguyen B.N., Wu R.C., White J., Deng C. (2017). Transforming growth factor-β in liver cancer stem cells and regeneration. Hepatol. Commun..

[131] Cong M., Iwaisako K., Jiang C., Kisseleva T. (2012). Cell signals influencing hepatic fibrosis. Int. J. Hepatol..

[132] Derynck R., Zhang Y.E. (2003). Smad-dependent and Smad-independent pathways in TGF-β family signalling. Nature.

[133] Li Q., Zhang D., Wang Y., Sun P., Hou X., Larner J., Xiong W., Mi J. (2013). MiR-21/Smad 7 signaling determines TGF-β1-induced CAF formation. Sci. Rep..

[134] Tsai W.C., Hsu P.W.C., Lai T.C., Chau G.Y., Lin C.W., Chen C.M., Lin C.D., Liao Y.L., Wang J.L., Chau Y.P. (2009). MicroRNA-122, a tumor suppressor microRNA that regulates intrahepatic metastasis of hepatocellular carcinoma. Hepatology.

[135] Li J., Ghazwani M., Zhang Y., Lu J., Li J., Fan J., Gandhi C.R., Li S. (2013). MiR-122 regulates collagen production via targeting hepatic stellate cells and suppressing P4HA1 expression. J. Hepatol..

[136] Bonner J.C. (2004). Regulation of PDGF and its receptors in fibrotic diseases. Cytokine Growth Factor Rev..

[137] Wang B., Li W., Guo K., Xiao Y., Wang Y., Fan J. (2012). MiR-181b promotes hepatic stellate cells proliferation by targeting p27 and is elevated in the serum of cirrhosis patients. Biochem. Biophys. Res. Commun..

[138] Meng F., Henson R., Wehbe–Janek H., Ghoshal K., Jacob S.T., Patel T. (2007). MicroRNA-21 regulates expression of the PTEN tumor suppressor gene in human hepatocellular cancer. Gastroenterology.

[139] Xie Y., Yao Q., Butt A.M., Guo J., Tian Z., Bao X., Li H., Meng Q., Lu J. (2014). Expression profiling of serum microRNA-101 in HBV-associated chronic hepatitis, liver cirrhosis, and hepatocellular carcinoma. Cancer Biol. Ther..

[140] Lei Y., Wang Q.-L., Shen L., Tao Y.-Y., Liu C.-H. (2019). MicroRNA-101 suppresses liver fibrosis by downregulating PI3K/Akt/mTOR signaling pathway. Clin. Res. Hepatol. Gastroenterol..

[141] Yang L., Dong C., Yang J., Yang L., Chang N., Qi C., Li L. (2019). MicroRNA-26b-5p inhibits mouse liver fibrogenesis and angiogenesis by targeting PDGF receptor-beta. Mol. Ther. Nucleic Acids.

[142] Wang X., Seo W., Park S.H., Fu Y., Hwang S., Rodrigues R.M., Feng D., Gao B., He Y. (2021). MicroRNA-223 restricts liver fibrosis by inhibiting the TAZ-IHH-GLI2 and PDGF signaling pathways via the crosstalk of multiple liver cell types. Int. J. Biol. Sci..

[143] Boulter L., Govaere O., Bird T.G., Radulescu S., Ramachandran P., Pellicoro A., Ridgway R.A., Seo S.S., Spee B., Van Rooijen N. (2012). Macrophage-derived Wnt opposes Notch signaling to specify hepatic progenitor cell fate in chronic liver disease. Nat. Med..

[144] Clevers H., Nusse R. (2012). Wnt/β-catenin signaling and disease. Cell.

[145] Miao C.-G., Yang Y.-Y., He X., Huang C., Huang Y., Zhang L., Lv X.-W., Jin Y., Li J. (2013). Wnt signaling in liver fibrosis: Progress, challenges and potential directions. Biochimie.

[146] Yu F., Fan X., Chen B., Dong P., Zheng J. (2016). Activation of hepatic stellate cells is inhibited by microRNA-378a-3p via Wnt10a. Cell. Physiol. Biochem..

[147] Liu H., Zhang S., Li Z., Zheng Z., Shi W., Hu M., Liu F. (2023). Promotion of hepatic stellate cell activation and liver fibrosis by microRNA-33a-5p through targeting the Dickkopf-1-mediated wingless-related integration site/beta-catenin pathway. J. Physiol. Pharmacol..

[148] Wang Q., Wei S., Li L., Bu Q., Zhou H., Su W., Liu Z., Wang M., Lu L. (2021). MiR-139-5p sponged by LncRNA NEAT1 regulates liver fibrosis via targeting β-catenin/SOX9/TGF-β1 pathway. Cell Death Discov..

[149] Kim N.H., Kim H.S., Kim N.-G., Lee I., Choi H.-S., Li X.-Y., Kang S.E., Cha S.Y., Ryu J.K., Na J.M. (2001). P53 and miRNA-34 are suppressors of canonical Wnt signaling. Sci. Signal..

[150] Tacke F., Luedde T., Trautwein C. (2009). Inflammatory pathways in liver homeostasis and liver injury. Clin. Rev. Allergy Immunol..

[151] Connolly E., Melegari M., Landgraf P., Tchaikovskaya T., Tennant B.C., Slagle B.L., Rogler L.E., Zavolan M., Tuschl T., Rogler C.E. (2008). Elevated expression of the miR-17–92 polycistron and miR-21 in hepadnavirus-associated hepatocellular carcinoma contributes to the malignant phenotype. Am. J. Pathol..

[152] Yu F., Guo Y., Chen B., Dong P., Zheng J. (2015). MicroRNA-17-5p activates hepatic stellate cells through targeting of Smad7. Lab. Investig..

[153] Noetel A., Kwiecinski M., Elfimova N., Huang J., Odenthal M. (2012). MicroRNA are central players in anti-and profibrotic gene regulation during liver fibrosis. Front. Physiol..

[154] Wu H.-C., Huang C.-L., Wang H.-W., Hsu W.-F., Tsai T.-Y., Chen S.-H., Peng C.-Y. (2019). Serum miR-21 correlates with the histological stage of chronic hepatitis B-associated liver fibrosis. Int. J. Clin. Exp. Pathol..

[155] You H., Wang X., Ma L., Zhang F., Zhang H., Wang Y., Pan X., Zheng K., Kong F., Tang R. (2023). Insights into the impact of hepatitis B virus on hepatic stellate cell activation. Cell Commun. Signal..

[156] Momen-Heravi F., Catalano D., Talis A., Szabo G., Bala S. (2021). Protective effect of LNA-anti-miR-132 therapy on liver fibrosis in mice. Mol. Ther.-Nucleic Acids.

[157] Cai Y., Huang G., Ma L., Dong L., Chen S., Shen X., Zhang S., Xue R., Sun D., Zhang S. (2018). Smurf2, an E3 ubiquitin ligase, interacts with PDE4B and attenuates liver fibrosis through miR-132 mediated CTGF inhibition. Biochim. Biophys. Acta (BBA)-Mol. Cell Res..

[158] Wei X., Tan C., Tang C., Ren G., Xiang T., Qiu Z., Liu R., Wu Z. (2013). Epigenetic repression of miR-132 expression by the hepatitis B virus x protein in hepatitis B virus-related hepatocellular carcinoma. Cell. Signal..

[159] Wang B., Hsu S.-H., Majumder S., Kutay H., Huang W., Jacob S.T., Ghoshal K. (2010). TGFβ-mediated upregulation of hepatic miR-181b promotes hepatocarcinogenesis by targeting TIMP3. Oncogene.

[160] Chen L., Charrier A., Zhou Y., Chen R., Yu B., Agarwal K., Tsukamoto H., Lee L.J., Paulaitis M.E., Brigstock D.R. (2014). Epigenetic regulation of connective tissue growth factor by MicroRNA-214 delivery in exosomes from mouse or human hepatic stellate cells. Hepatology.

[161] Izawa T., Horiuchi T., Atarashi M., Kuwamura M., Yamate J. (2015). Anti-fibrotic role of miR-214 in thioacetamide-induced liver cirrhosis in rats. Toxicol. Pathol..

[162] Shen W.-J., Dong R., Chen G., Zheng S. (2014). MicroRNA-222 modulates liver fibrosis in a murine model of biliary atresia. Biochem. Biophys. Res. Commun..

[163] Tao L., Xue D., Shen D., Ma W., Zhang J., Wang X., Zhang W., Wu L., Pan K., Yang Y. (2018). MicroRNA-942 mediates hepatic stellate cell activation by regulating BAMBI expression in human liver fibrosis. Arch. Toxicol..

[164] Zhou L., Liu S., Han M., Ma Y., Feng S., Zhao J., Lu H., Yuan X., Cheng J. (2018). MiR-185 Inhibits Fibrogenic Activation of Hepatic Stellate Cells and Prevents Liver Fibrosis. Mol. Ther.-Nucleic Acids.

[165] Li B.-B., Li D.-L., Chen C., Liu B.-H., Xia C.-Y., Wu H.-J., Wu C.-Q., Ji G.-Q., Liu S., Ni W. (2016). Potentials of the elevated circulating miR-185 level as a biomarker for early diagnosis of HBV-related liver fibrosis. Sci. Rep..

[166] Brea R., Motiño O., Francés D., García-Monzón C., Vargas J., Fernández-Velasco M., Boscá L., Casado M., Martín-Sanz P., Agra N. (2018). PGE2 induces apoptosis of hepatic stellate cells and attenuates liver fibrosis in mice by downregulating miR-23a-5p and miR-28a-5p. Biochim. Biophys. Acta (BBA)-Mol. Basis Dis..

[167] Wan L.-Y., Peng H., Ni Y.-R., Jiang X.-P., Wang J.-J., Zhang Y.-Q., Ma L., Li R., Han L., Tan Y. (2022). The miR-23b/27b/24-1 Cluster Inhibits Hepatic Fibrosis by Inactivating Hepatic Stellate Cells. Cell. Mol. Gastroenterol. Hepatol..

[168] Zeng X.-Y., Zhang Y.-Q., He X.-M., Wan L.-Y., Wang H., Ni Y.-R., Wang J., Wu J.-F., Liu C.-B. (2016). Suppression of hepatic stellate cell activation through downregulation of gremlin1 expression by the miR-23b/27b cluster. Oncotarget.

[169] Zhang J., Zhang H., Liu J., Tu X., Zang Y., Zhu J., Chen J., Dong L., Zhang J. (2012). MiR-30 inhibits TGF-β1-induced epithelial-to-mesenchymal transition in hepatocyte by targeting Snail1. Biochem. Biophys. Res. Commun..

[170] Tsai W.-C., Hsu S.-D., Hsu C.-S., Lai T.-C., Chen S.-J., Shen R., Huang Y., Chen H.-C., Lee C.-H., Tsai T.-F. (2012). MicroRNA-122 plays a critical role in liver homeostasis and hepatocarcinogenesis. J. Clin. Investig..

[171] Yang Z., Peng Y., Yang S. (2019). MicroRNA-146a regulates the transformation from liver fibrosis to cirrhosis in patients with hepatitis B via interleukin-6. Exp. Ther. Med..

[172] Liao Z., Zheng R., Shao G. (2022). Mechanisms and application strategies of miRNA-146a regulating inflammation and fibrosis at molecular and cellular levels. Int. J. Mol. Med..

[173] Jung K.H., Zhang J., Zhou C., Shen H., Gagea M., Rodriguez-Aguayo C., Lopez-Berestein G., Sood A.K., Beretta L. (2016). Differentiation therapy for hepatocellular carcinoma: Multifaceted effects of miR-148a on tumor growth and phenotype and liver fibrosis. Hepatology.

[174] Venugopal S.K., Jiang J., Kim T.-H., Li Y., Wang S.-S., Torok N.J., Wu J., Zern M.A. (2009). Liver fibrosis causes downregulation of miRNA-150 and miRNA-194 in hepatic stellate cells, and their overexpression causes decreased stellate cell activation. Am. J. Physiol.-Gastrointest. Liver Physiol..

[175] Li L., Zhang L., Zhao X., Cao J., Li J., Chu G. (2019). Downregulation of miR-152 contributes to the progression of liver fibrosis via targeting Gli3 in vivo and in vitro. Exp. Ther. Med..

[176] Hyun J., Wang S., Kim J., Rao K.M., Park S.Y., Chung I., Ha C.-S., Kim S.-W., Yun Y.H., Jung Y. (2016). MicroRNA-378 limits activation of hepatic stellate cells and liver fibrosis by suppressing Gli3 expression. Nat. Commun..

[177] Morris S.M., Baek J.Y., Koszarek A., Kanngurn S., Knoblaugh S.E., Grady W.M. (2012). Transforming growth factor-beta signaling promotes hepatocarcinogenesis induced by p53 loss. Hepatology.

[178] Pineau P., Volinia S., McJunkin K., Marchio A., Battiston C., Terris B., Mazzaferro V., Lowe S.W., Croce C.M., Dejean A. (2010). MiR-221 overexpression contributes to liver tumorigenesis. Proc. Natl. Acad. Sci. USA.

[179] Roy S., Benz F., Luedde T., Roderburg C. (2015). The role of miRNAs in the regulation of inflammatory processes during hepatofibrogenesis. Hepatobiliary Surg. Nutr..

[180] Liu G., Friggeri A., Yang Y., Milosevic J., Ding Q., Thannickal V.J., Kaminski N., Abraham E. (2010). MiR-21 mediates fibrogenic activation of pulmonary fibroblasts and lung fibrosis. J. Exp. Med..

[181] Zhao H., Wu L., Yan G., Chen Y., Zhou M., Wu Y., Li Y. (2021). Inflammation and tumor progression: Signaling pathways and targeted intervention. Signal Transduct. Target. Ther..

[182] Hu T., Zhang W., Xu C. (2023). Expression of miR-223-3p in patients with hepatitis B virus liver fibrosis and its effect on hepatic stellate cells: An observational study. Medicine.

[183] Luo X., Zhang R., Lu M., Liu S., Baba H.A., Gerken G., Wedemeyer H., Broering R. (2021). Hippo pathway counter-regulates innate immunity in hepatitis B virus infection. Front. Immunol..

[184] Zanconato F., Cordenonsi M., Piccolo S. (2016). YAP/TAZ at the roots of cancer. Cancer Cell.

[185] Bao S., Zheng J., Li N., Huang C., Chen M., Cheng Q., Yu K., Chen S., Zhu M., Shi G. (2017). Serum MicroRNA Levels as a Noninvasive Diagnostic Biomarker for the Early Diagnosis of Hepatitis B Virus-Related Liver Fibrosis. Gut Liver.

[186] Jiang X.-P., Ai W.-B., Wan L.-Y., Zhang Y.-Q., Wu J.-F. (2017). The roles of microRNA families in hepatic fibrosis. Cell Biosci..

[187] Su J., Zhang A., Shi Z., Ma F., Pu P., Wang T., Zhang J., Kang C., Zhang Q. (2012). MicroRNA-200a suppresses the Wnt/β-catenin signaling pathway by interacting with β-catenin. Int. J. Oncol..

[188] Zhou D.-D., Wang X., Wang Y., Xiang X.-J., Liang Z.-C., Zhou Y., Xu A., Bi C.-H., Zhang L. (2016). MicroRNA-145 inhibits hepatic stellate cell activation and proliferation by targeting ZEB2 through Wnt/β-catenin pathway. Mol. Immunol..

[189] Pathania A.S., Bhat S.A., Adepoju L.A., Kharbanda K.K., Osna N.A. (2025). Hepatic Macrophages in Chronic Hepatitis B: Balancing Immunity and Pathology. Biology.

[190] Li A., Yi Z., Ma C., Sun B., Zhao L., Cheng X., Hui L., Xia Y. (2025). Innate immune recognition in hepatitis B virus infection. Virulence.

[191] Chaparro M., Sanz-Cameno P., Trapero-Marugán M., García-Buey L., Moreno-Otero R. (2007). Mechanisms of angiogenesis in chronic inflammatory liver disease. Ann. Hepatol..

[192] Yu K., Shi G., Li N. (2015). The function of MicroRNA in hepatitis B virus-related liver diseases: From Dim to Bright. Ann. Hepatol..

[193] Li C.H., Xu F., Chow S., Feng L., Yin D., Ng T.B., Chen Y. (2014). Hepatitis B virus X protein promotes hepatocellular carcinoma transformation through interleukin-6 activation of microRNA-21 expression. Eur. J. Cancer.

[194] Jia C.-C., Wang T.-T., Liu W., Fu B.-S., Hua X., Wang G.-Y., Li T.-J., Li X., Wu X.-Y., Tai Y. (2013). Cancer-associated fibroblasts from hepatocellular carcinoma promote malignant cell proliferation by HGF secretion. PLoS ONE.

[195] Ali E., Trailin A., Ambrozkiewicz F., Liška V., Hemminki K. (2022). Activated hepatic stellate cells in hepatocellular carcinoma: Their role as a potential target for future therapies. Int. J. Mol. Sci..

[196] Yuan X., Huang C., Ran Y. (2025). Exosome in HBV infection: Current concepts and future perspectives. Front. Cell. Infect. Microbiol..

[197] Zhou Y., Ren H., Dai B., Li J., Shang L., Huang J., Shi X. (2018). Hepatocellular carcinoma-derived exosomal miRNA-21 contributes to tumor progression by converting hepatocyte stellate cells to cancer-associated fibroblasts. J. Exp. Clin. Cancer Res..

[198] Santana-Viera L., Ibba M.L., Rotoli D., Catuogno S., Esposito C.L. (2020). Emerging therapeutic RNAs for the targeting of cancer associated fibroblasts. Cancers.

[199] Sharip A., Kunz J. (2025). Mechanosignaling via integrins: Pivotal players in liver fibrosis progression and therapy. Cells.

[200] Papait A., Romoli J., Stefani F.R., Chiodelli P., Montresor M.C., Agoni L., Silini A.R., Parolini O. (2022). Fight the cancer, hit the CAF!. Cancers.

[201] Gu W.J., Shen Y.W., Zhang L.J., Zhang H., Nagle D.G., Luan X., Liu S.H. (2021). The multifaceted involvement of exosomes in tumor progression: Induction and inhibition. MedComm.

[202] Saadh M.J., Jasim N.Y., Ahmed M.H., Ballal S., Kumar A., Atteri S., Vashishth R., Rizaev J., Alhili A., Jawad M.J. (2025). Critical roles of miR-21 in promotions angiogenesis: Friend or foe?. Clin. Exp. Med..

[203] Shi X., Zhu W., Zhang J., Fan C., Zhang J. (2025). Cancer-associated fibroblasts in hepatocellular carcinoma: Origins, heterogeneity, and therapeutic implications. Front. Immunol..

[204] Jia W., Liang S., Cheng B., Ling C. (2021). The role of cancer-associated fibroblasts in hepatocellular carcinoma and the value of traditional Chinese medicine treatment. Front. Oncol..

[205] Cadamuro M., Nuozzi G., Simioni P., Fabris L. (2023). The tumor microenvironment in hepatocarcinoma: Dissecting the functions of cancer-associated fibroblasts. Hepatoma Res..

[206] Miaomiao S., Xiaoqian W., Yuwei S., Chao C., Chenbo Y., Yinghao L., Yichen H., Jiao S., Kuisheng C. (2023). Cancer-associated fibroblast-derived exosome microRNA-21 promotes angiogenesis in multiple myeloma. Sci. Rep..

