# Supplementary material for: MicroRNA Control of Hepatocyte–Stromal Crosstalk in the Early Premalignant Microenvironment of HBV-Associated HCC
Source: Int J Mol Sci. 2026 Aug 24;27(17):7581. doi: 10.3390/ijms27177581 (PMC13566340; doi:10.3390/ijms27177581)
Supplement: Supplementary file 1 [file ijms-27-07581-s001.zip › ijms-4499072-supplementary.pdf]

Supplementary Table S1: Validation of HBV versus non-HBV- miRNA fibrosis models

miR-17-5p: Pro-fibrotic; targets WIF1 and SMAD7; promotes Wnt/beta-catenin signaling and HSC activation | non-HBV fibrosis/HSC evidence; HBV/hepadnaviral association is primarily oncogenic | Experimental HSC activation and general liver-fibrosis studies; hepadnaviral-associated HCC. Plausible pro-fibrotic mechanism but not directly validated as an HBV-induced fibrosis driver (43-45).

miR-21-5p: Pro-fibrotic; represses SMAD7 and PTEN; amplifies TGF-beta/SMAD and PI3K/AKT signaling. Direct HBV-associated evidence plus broader non-HBV mechanistic evidence | HBV-associated fibrosis clinical correlation; HBV/HBx-related systems; experimental fibrosis/HSC studies | Strong HBV-relevant candidate; direct HBV-specific functional validation should nevertheless be distinguished from general fibrosis mechanisms | 46-49; see also main-text refs (31, 77, 89, 91).

miR-27a: Pro-fibrotic; associated with HSC activation and collagen expression; reported targets include PPAR-gamma, FOXO1, APC and p53 | HBV-associated clinical/HSC evidence | HBV-induced cirrhosis and HSC activation study | One of the stronger HBV-associated entries; clinical association supports relevance, although further causal HBV-model validation remains desirable (50).

|**miR-33a**: Pro-fibrotic; promotes Akt-associated HSC activation; reported links to SMAD7 and PPAR pathways | HBV-associated clinical evidence plus non-HBV mechanistic HSC evidence | Hepatic and serum samples from chronic HBV-induced fibrosis; HSC studies | HBV-associated expression evidence is available; mechanistic pathway assignment is not exclusively HBV-specific (51-53).

miR-132: Context-dependent; regulates SIRT1/CTGF-related fibrogenic programs. HBx-associated dysregulation; fibrosis function mainly non-HBV. HBx/HBV-HCC study; mouse fibrosis and non-HBV mechanistic studies. HBx can repress miR-132, but its anti-/pro-fibrotic role should not be presented as directly validated in HBV fibrosis (54-56).

miR-181b: Pro-fibrotic; targets p27 and PTEN; promotes HSC proliferation and activation. non-HBV HSC/cirrhosis evidence HSC cultures and cirrhosis-associated serum studies | Relevant to liver fibrogenesis, but direct HBV-induced fibrosis validation is lacking in the cited studies | 57-59

miR-214-5p: Generally anti-fibrotic; regulates CTGF and TGF-beta-associated pathways; can promote HSC apoptosis. non-HBV experimental fibrosis evidence. Mouse/human HSC exosome studies and thioacetamide-induced cirrhosis in rats. Mechanistically relevant but not validated in an HBV-induced fibrosis model (60, 61).

miR-222 | Pro-fibrotic; promotes HSC activation and survival; HBV-infected hepatocyte exosomes act through the miR-222/TFRC axis | Direct HBV-associated evidence | HBV-infected hepatocyte-derived exosomes; additional biliary-atresia fibrosis model | Strong direct evidence for HBV-conditioned hepatocyte-to-HSC communication; one of the best examples of HBV-specific crosstalk (62-64).

miR-942| Pro-fibrotic; targets BAMBI and promotes HSC activation | non-HBV human fibrosis/HSC evidence | Human liver-fibrosis and HSC activation studies | Supports a general fibrogenic mechanism; no direct HBV-specific validation in the cited evidence (51, 65).

miR-18a | Reduced miR-18a can increase CTGF-associated fibrogenic signaling | HBx-associated CTGF evidence; primary context is HBV-associated hepatocarcinoma | HBx-expressing or HBV-associated HCC systems | Relevant to an HBx–CTGF axis, but stronger for HBV-HCC biology than established HBV-fibrosis models (51, 66).

miR-19b | Anti-fibrotic; suppresses TGF-beta/GRB2-related HSC fibrogenesis and collagen expression | non-HBV HSC evidence | Experimental HSC-mediated fibrogenesis | General anti-fibrotic mechanism; requires direct validation in HBV-induced fibrosis (67).

miR-185 | Anti-fibrotic; restrains RICTOR/RHEB/SREBF1-linked HSC activation | HBV clinical biomarker association; functional evidence mainly non-HBV | Experimental HSC/liver-fibrosis studies; circulating miR-185 in HBV-related fibrosis | Potential HBV fibrosis biomarker, but the proposed anti-fibrotic mechanism is not specifically validated in HBV models (51, 68, 69).

miR-23a-5p | Context-dependent; affects ERK, BMP2, CAV-1 and CDH1; linked to HSC apoptosis | non-HBV experimental fibrosis evidence | Mouse experimental fibrosis and HSC studies | Not directly validated in HBV-induced fibrosis (70).

miR-23b/miR-27b | Anti-fibrotic; regulates LOX, CTGF, TIMP1 and Gremlin1; suppresses HSC activation | non-HBV HSC/fibrosis evidence | Experimental HSC activation and general fibrosis studies | General anti-fibrotic pathway; direct HBV fibrosis evidence is absent in the cited studies (71, 72).

miR-29b | Anti-fibrotic; suppresses TGF-beta/PI3K/AKT-associated HSC activation and collagen/TIMP-1 expression | HBV clinical association; functional evidence mainly non-HBV | HBV patient serum/liver association; human/murine fibrosis and HSC models | Strong HBV disease association and strong general fibrosis evidence, but the central functional studies are not HBV-specific (38, 73, 74).

miR-29a | Anti-fibrotic; represses IGF-1, PDGF and ECM/collagen-related targets | HBV clinical association; functional evidence mainly non-HBV | HBV patient association; HSC and general fibrosis studies | Important HBV-relevant candidate; avoid describing the detailed PDGF/ECM mechanism as HBV-model validated (38, 46, 75).

miR-30 | Anti-fibrotic/anti-EMT; targets SNAIL1 in TGF-beta-induced EMT | non-HBV hepatocyte EMT/fibrosis evidence | TGF-beta-stimulated hepatocyte EMT model | General mechanistic evidence only; distinguished from the HBx-specific miR-30e entry (76).

miR-30e | Reduced miR-30e promotes fibrosis through P4HA2 | Direct HBx-associated fibrosis evidence | HBx-related liver fibrosis and hepatoma model | Strong HBx-associated candidate supporting a direct viral miRNA-dysregulation pathway (77).

miR-34a-5p | Context-dependent; reported to regulate TGF-beta1/SMAD3 and NF-kappaB/HMGB1/SMAD4-related processes | non-HBV fibrosis/HSC evidence; direction differs among studies | General HSC and fibrosis models | Do not assign a uniform direction or claim HBV-specific validation; evidence is context-dependent (51, 78).

miR-101b | Anti-fibrotic; suppresses TGF-beta/PI3K/AKT/mTOR-associated HSC activation | HBV clinical association; functional evidence mainly non-HBV | Serum profiling in HBV chronic hepatitis, cirrhosis and HCC; general fibrosis studies | Potential HBV biomarker; causal anti-fibrotic mechanism needs HBV-model confirmation (79, 80).

miR-122 | Liver-enriched, generally protective; regulates TGFB1, KLF6 and P4HA1 and restrains collagen production | HBV/HBx-associated dysregulation; HSC mechanism mainly non-HBV | HBV patient/HBx studies; HSC and liver homeostasis models | Strongly relevant to HBV pathogenesis, but the collagen/P4HA1 mechanism should be described as non-HBV-model evidence unless separately validated (81, 82); see also main-text (20, 21, 78).

miR-146a | Context-dependent inflammation/fibrosis regulator; linked to IL-6 and SMAD4 pathways | HBV-associated clinical evidence; broader mechanism mainly non-HBV | HBV patient fibrosis-to-cirrhosis study; general inflammation/fibrosis literature | Supports HBV disease association, but direct mechanistic HBV-fibrosis validation remains limited (83, 84)

miR-148a | Anti-fibrotic/tumour-suppressive candidate; regulates I-kappaB-alpha, NUMB and NOTCH | non-HBV fibrosis/HCC evidence | HCC and liver fibrosis differentiation-therapy model | Relevant pathway-level evidence but not an HBV-specific fibrosis mechanism in the cited study (85).

miR-200a: Context-dependent regulator of TGF-beta-mediated HSC activation and beta-catenin signaling | non-HBV TGF-beta-stimulated HSC evidence TGF-beta1-mediated HSC activation model | Relevant to epithelial/stromal plasticity, but not directly validated as an HBV-induced fibrosis mechanism (89).

miR-150 | Anti-fibrotic; regulates c-Myb and Rac1 and restrains alpha-SMA/COL1A1 expression | non-HBV HSC/fibrosis evidence | General liver-fibrosis and HSC studies | Not directly validated in HBV-induced fibrosis (86).

miR-152/miR-378a: Anti-fibrotic; suppresses Gli3-associated HSC activation and collagen expression | non-HBV fibrosis evidence | In vivo and in vitro experimental fibrosis/HSC models | General anti-fibrotic evidence; no direct HBV-specific validation in the cited studies (87, 88).

Abbreviations: AKT, protein kinase B; BAMBI, BMP and activin membrane-bound inhibitor; CTGF, connective tissue growth factor; ECM, extracellular matrix; HBV, hepatitis B virus; HBx, hepatitis B virus X protein; HSC, hepatic stellate cell; PI3K, phosphoinositide 3-kinase; PTEN, phosphatase and tensin homolog; SMAD, suppressor of mothers against decapentaplegic; TGF-beta, transforming growth factor-beta; TFRC, transferrin receptor.

Notes: Direct HBV-associated evidence includes HBV-infected or HBx-expressing experimental systems, HBV-associated patient samples, or HBV-infected hepatocyte-derived extracellular vesicles. HBV clinical association refers to expression or biomarker studies in CHB, HBV-related fibrosis, cirrhosis, or HBV-associated HCC without a direct HBV fibrosis mechanism. Non-HBV fibrosis evidence includes CCl4, thioacetamide, bile-duct-ligation, metabolic, generic HSC-activation, or other non-HBV liver-fibrosis models. These studies are mechanistically informative but require direct validation in HBV-specific fibrosis models.
